# No evidence for contextual cueing beyond explicit recognition

**DOI:** 10.3758/s13423-023-02358-3

**Published:** 2023-10-16

**Authors:** Sascha Meyen, Miguel A. Vadillo, Ulrike von Luxburg, Volker H. Franz

**Affiliations:** 1 Department of Computer Science, University of Tübingen, Sand 6, 72076 Tübingen, Germany; 2 Universidad Autónoma de Madrid, Madrid, Spain; 3 Tübingen AI Center, Tübingen, Germany

**Keywords:** Implicit/explicit memory, Contextual cueing, Indirect task advantage, Signal detection theory

## Abstract

Many studies claim that visual regularities can be learned unconsciously and without explicit awareness. For example in the contextual cueing paradigm, studies often make claims using a *standard reasoning* based on two results: (1) a reliable response time (RT) difference between repeated vs. new stimulus displays and (2) a close-to-chance sensitivity when participants are asked to explicitly recognize repeated stimulus displays. From this pattern of results, studies routinely conclude that the sensitivity of RT responses is higher than that of explicit responses—an empirical situation we call *Indirect Task Advantage* (ITA). Many studies further infer from an ITA that RT effects were driven by a form of recognition that exceeds explicit memory: *implicit recognition*. However, this reasoning is flawed because the sensitivity underlying RT effects is never computed. To properly establish a difference, a *sensitivity comparison* is required. We apply this sensitivity comparison in a reanalysis of 20 contextual cueing studies showing that not a single study provides consistent evidence for ITAs. Responding to recent correlation-based arguments, we also demonstrate the absence of evidence for ITAs at the level of individual participants. This lack of ITAs has serious consequences for the field: If RT effects can be fully explained by weak but above-chance explicit recognition sensitivity, what is the empirical content of the label “implicit”? Thus, theoretical discussions in this paradigm-and likely in other paradigms using this standard reasoning-require serious reassessment because the current data from contextual cueing studies is insufficient to consider recognition as implicit.

**Supplementary Information:**

The online version contains supplementary material available at 10.3758/s13423-023-02358-3.

How do humans learn regularities in the continual stream of visual input? The literature holds that visual regularities can be learned *implicitly* and without awareness (Frost et al., 2019; Goujon et al., 2015; Jiang , 2018; Theeuwes et al., 2022; Turk-Browne et al., 2005). There are several paradigms supporting this notion of which the *contextual cueing* paradigm is one of the most prominent ones (Chun & Jiang, 1998; Chun, 2000; Chun & Jiang, 2003; Jiang & Sisk, 2019).

Whether or not contextual cueing occurs implicitly has been controversially debated since its inception (Cleeremans et al., 1998; Colagiuri and Livesey , 2016; Kroell et al., 2019; Geyer et al., 2010; Geyer et al., 2012; Geyer et al., 2020; Goldstein et al., 2022; Schlagbauer et al., 2012; Smyth & Shanks, 2008; Sisk et al., 2019; Vadillo et al., 2016; Vadillo et al., 2022). Here, we add a new argument to this debate by scrutinizing the frequently used but flawed *standard reasoning* for inferring implicit recognition. This standard reasoning seeks to demonstrate that indirect measures of recognition (such as RT effects) reveal a higher sensitivity than participants’ explicit reports—a purely empirical condition that we call the *Indirect Task Advantage* (ITA). Together with further assumptions, such an ITA is typically interpreted as indicating some form of recognition that exceeds explicit recognition and is, therefore, considered evidence for implicit recognition. But we will show that the standard reasoning fails to properly establish evidence for an ITA for methodological reasons.

As a replacement, we describe the appropriate method to properly establish an ITA: the *sensitivity comparison*. In a preregistered reanalysis of 20 studies, we investigate the evidence of ITAs with this more appropriate method and show that there is little to no evidence for ITAs in those studies.

Given this striking lack of evidence for ITAs in studies that have previously inferred implicit recognition from their data, it is plausible that weak explicit recognition drives contextual cueing RT effects. Without an ITA, there is no evidence for recognition beyond what participants can explicitly report. Therefore, strong claims about recognition being implicit require more evidence than what is currently presented. Our finding has serious implications for theoretical reasoning about contextual cueing effects and their implicit vs. explicit nature.

Our critique is similar to that brought forward in the *masked priming* paradigm where an analogous problem occurred (Franz & von Luxburg, 2015; Meyen et al., 2022; Schnepf et al., 2022; Zerweck et al. , 2021). This shows that the flaw in the standard reasoning is pervasive and likely generalizes to many other paradigms. Overall, we conclude that the label “implicit” or “unconscious” should be used more cautiously.

## The standard reasoning is used to infer an indirect task advantage

**Fig. 1:**
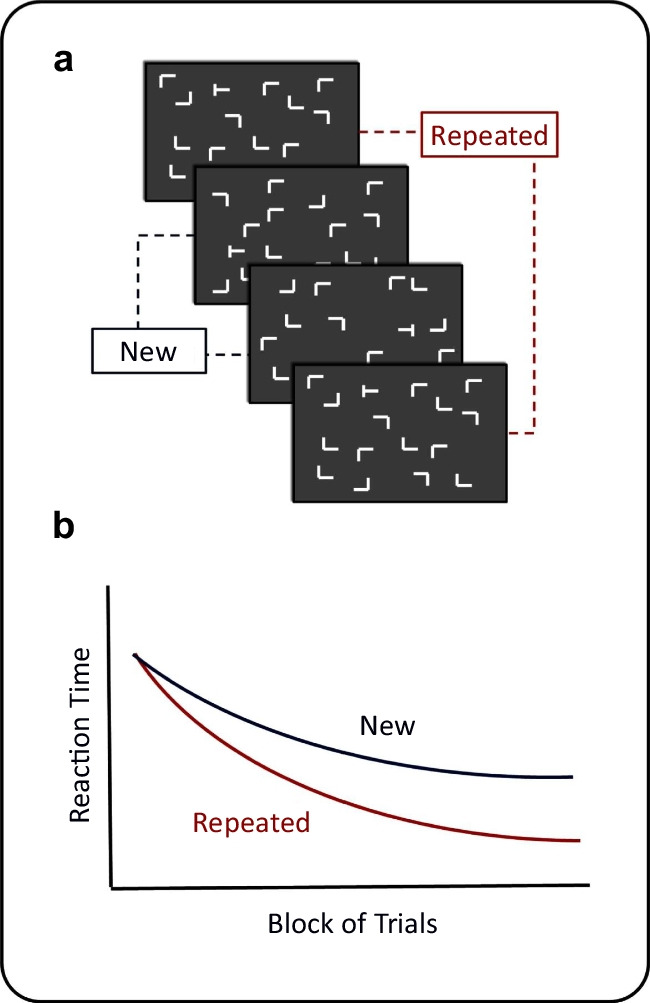
Contextual Cueing Search Task. (a) In the search task of contextual cueing experiments, participants see a display consisting of a configurations of distractors (Ls) and a target stimulus (T) that they have to find. (b) Over the course of the experiment, participants produce faster RTs for stimulus configurations that are repeated throughout the experiment vs. those that are randomly generated anew in each trial. This demonstrates that there is some recognition of the repeated configurations. Figure adapted from Vadillo et al. (2016), used under the http://creativecommons.org/licenses/by/4.0/Creative Commons Attribution 4.0 International License

Implicit recognition is typically inferred from two tasks yielding the following pattern of results. In a *search task* (see Fig. 1a), participants are presented with displays of stimulus configurations and their task is to find the one odd stimulus. Most commonly, participants search for a T among Ls. They do this in hundreds of trials over the course of the experiment. In half of these trials, a small number of stimulus configurations are repeated over and over such that participants have the chance to recognize these configurations throughout the experiment. In the other half of the trials, the stimulus configurations are always randomly generated anew so that no recognition is possible. As a result, participants show a search time advantage (see diverging curves in Fig. 1b): Over the course of the experiment, RTs become faster for repeated configurations than for the new configurations. This indicates that participants recognized the repeated configurations at some level allowing them to find the odd stimulus faster.

**Fig. 2:**
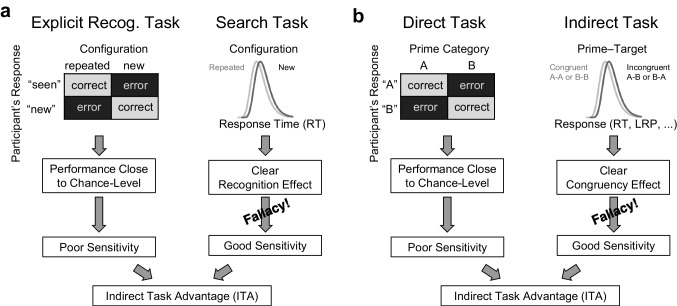
Flawed Standard Reasoning to Infer an Indirect Task Advantage (ITA). (a) The standard reasoning to infer an ITA in contextual cueing is based on two tasks. In the search task (right strand), a clear RT effect between repeated vs. new stimulus configurations indicates that participants have recognized the repeated configurations at some level. In the explicit recognition task (left strand), participants’ explicit report sensitivity is typically close to chance level. From this, the standard reasoning erroneously infers a higher sensitivity underlying search task responses as compared with explicit recognition task responses; it infers an ITA. Based on this apparent ITA and with additional theoretical assumptions (not shown in the figure), recognition in the search task is inferred to be implicit. (b) The analogous reasoning is prevalent in masked priming, where a clear congruency effect (right strand) together with a close-to-chance-level sensitivity in detecting the prime stimulus (left strand) is often erroneously thought to be evidence for an ITA and, further, to be evidence for unconscious processing of the prime. Figure adapted from Meyen et al. (2022).

Was this recognition implicit or explicit? To answer this question, an *explicit recognition task* is added at the end of the experiment. There are multiple variants of this task. The most common variant is to show participants one configuration per trial and ask them whether they recognize this configuration as a repeated one from the previous search task. The performance in these binary explicit recognition tasks is measured in terms of signal detection theory’s sensitivity $d'$. This is typically low and close to chance level ($d' < 0.3$) indicating at most weak explicit recognition of the repeated configurations.

In another common variant, participants see two stimulus configurations per trial: One was repeated and one is new. They then have to choose the configuration from the two that they believe was repeated throughout the search task. Again, sensitivity $d'$ is calculated to evaluate participants’ explicit recognition.

Both of these variants ask for binary responses. In contrast, there are other, less common variants in which participants have to guess the location of the target stimulus (see Jiang & Chun, 2003, and Smyth & Shanks, 2008 for an overview). In such tasks, participants are shown an altered stimulus configuration in which the odd stimulus (T) is replaced by a regular stimulus (L). Then, participants have to guess in which position (or, typically, in which quadrant) the odd stimulus was in the originally displayed configuration. This task requires responses that are not binary and are more difficult to relate to the RT data. For this reason, we will focus on the much more frequently employed explicit recognition tasks with binary responses.

Together with the clear RT effects in the search task, the *standard reasoning* incorrectly infers implicit recognition (see Fig. 2 for an illustration): Participants’ explicit recognition was only weak but the clear RT effects seem to indicate better recognition, thus, there seems to be evidence for recognition beyond what participants can explicitly report—implicit recognition. In short, a weak explicit recognition task performance together with a clear RT effect has been the typical basis for inferring implicit recognition.

This standard reasoning in contextual cueing is analogous to that used in the unconscious priming paradigm Eriksen , 1960; Holender , 1986; Simons et al., 2007), where a clear priming effect on RTs (or other indirect measures) combined with a weak direct identification performance of the primes is often taken as evidence for priming being *unconscious* (see Fig. 2b). We started our investigation with the priming paradigm where we introduced the term Indirect Task Advantage (ITA; Meyen et al., 2022) and we continue using this term here for consistency. If we had started in the contextual cueing paradigm, we could have called it the *Implicit Task Advantage* instead. With the standard reasoning and its crucial component—the ITA—outlined, we proceed by explaining its flaw.

## The standard reasoning produces unwarranted ITA conclusions

*The main problem of the standard reasoning is that the same source of (weak explicit) recognition can produce the typical pattern of results in search and explicit recognition tasks*. The two tasks measure two very different things: mean RT effects in the search task vs. discrimination sensitivities at the single-trial level in the explicit recognition task. These two measures are not comparable (unless adequate methods are used) and one cannot conclude a difference in underlying sensitivities—an ITA—based on such results.

Nevertheless, this is the standard reasoning that is used not only in contextual cueing but also in other paradigms such as the probabilistic cueing paradigm (Jiang et al., 2018; Jiang et al., 2013) and the additional singleton paradigm (Wang & Theeuwes, 2018). But, as demonstrated in previous research by Franz and von Luxburg (2015) and Meyen et al. (2022), the standard reasoning produced misleading interpretations. These misleading interpretations occurred, unfortunately, despite the problems of the standard reasoning being theoretically discussed throughout the past decades (Eriksen, 1960; Reingold & Merikle, 1990; Reingold, 2004).

The fallacy of the standard reasoning is especially alluring in contextual cueing because RT effects are typically very large. They range from 50 ms (Chun & Jiang, 1998) up to 466 ms (Zang et al., 2015). These RT effects appear very strong when contrasted with the weak explicit recognition task sensitivity, which typically ranges from $d' = 0.1$ to $d' = 0.3$ (corresponding to 52–56%-correct, see Section “Reanalysis of 20 Contextual Cueing Studies”). This creates a misleading impression of a difference in sensitivities underlying the two tasks.

But the large RT effects are a consequence of the search task having long overall RTs. In contextual cueing, participants have to make multiple fixations as opposed to priming where presentation conditions are much more limited. As a result, overall RTs as well as RT effects are scaled up. For example, Zang et al. (2015)’s huge 466 ms RT effect occurred in a condition with an overall mean RT of around 2500 ms. Crucially, the long overall RTs are accompanied by larger trial-by-trial standard deviations putting large RT effects into perspective (see Miller & Ulrich, 2013, for a related argument in other paradigms). In contextual cueing, participants have to search through many stimuli in the configurations. In some trials, they are lucky and find the target stimulus very fast. But in other trials, they have to search through almost the entire stimulus configuration before finding the target stimulus. This leads to a large variability in RTs from trial to trial. This large trial-by-trial variability then attenuates the large absolute RT effects: Relative effects such as the sensitivity—which is here the absolute RT effect divided by the trial-by-trial variability—may therefore be surprisingly low and even comparable to the weak sensitivity measured in the explicit recognition task. This does not contradict the fact that these effects are very reliably found in contextual cueing studies even with few number of participants, because studies employ many hundreds of trials in the search tasks thereby ameliorating the impact of trial-by-trial noise.

But if the sensitivity underlying the RT effects were as weak as observed in the explicit recognition task, there would be no evidence for recognition that goes beyond what participants can explicitly report. Without an ITA there would be no evidence for a second source of (implicit) recognition. The following intuitive example serves to illustrate this fallacy of the standard reasoning.

### Illustration of the flaw: Baby-weight example

Consider a mock-experiment to illustrate the problem of the standard reasoning (adapted from Meyen et al., 2022). In this mock-experiment, participants measure the body weights of newborn baby girls and boys. In an indirect task trial, a participant measures a baby’s weight and the observed response is just that measured weight (corresponding to a single-trial RT in the search task). A researcher evaluating weight responses over many hundreds of trials will find a clear absolute weight difference between baby girls and baby boys of around 100 g (Zeitlin et al., 2017). This corresponds to the typical result in the search task where a clear and reliable RT difference of sometimes around 100 ms is found.

In a direct task trial, a participant again measures the weight of a baby but this time they has to judge whether this baby is a girl or a boy (corresponding to the binary responses required in the explicit recognition task). The biological sex is hidden during the weight measurement so that the participant has to make a guess solely based on the measured weight. Even though there is a clear mean difference of 100 g, the variability of individual babies’ body weight is substantial: With a trial-by-trial standard deviation of approximately 400 g (Zeitlin et al., 2017), weight distributions heavily overlap and participants’ performance will be close to chance level: a sensitivity of $d' = \frac{100~g}{400~g} = 0.25$ (where chance level is $d'= 0$). Even if participants apply the optimal, unbiased decision criterion, hit rates (proportion of baby girls correctly classified as girls) and false alarm rates (proportion of baby boys incorrectly classified as girls) would be around $HR = 55\%$ and $FA = 45\%$ corresponding to an overall accuracy of 55%. Biased observers would show even lower accuracies.

This is the typical pattern of results from which the standard reasoning would infer implicit recognition (of the baby’s sex) even though, in this thought experiment, participants have full explicit information in both tasks (i.e., the body weight of the babies). In one task, the weight information produces a clear mean effect, while in the other task it produces a low sensitivity. One should therefore not infer a difference based on this pattern of results. Researchers must rule out the possibility that responses in both tasks can be explained by the same (weak) sensitivity. Otherwise, claims about different processes, for example, explicit vs. implicit recognition may be incorrect.

## The appropriate method: Sensitivity comparison

We argue that the appropriate analysis to provide evidence for implicit recognition requires a *sensitivity comparison* (Meyen et al., 2022; called *relative sensitivity paradigm* by Merikle & Reingold, 1991; Reingold , 2004; see also Eriksen , 1960): The first step of inferring implicit recognition is to convincingly demonstrate that RTs in the search task are more sensitive than responses in the explicit recognition task—that is, an ITA must be empirically established. Such an ITA would represent “excess” sensitivity in the search task that goes beyond the sensitivity in participants’ explicit responses.

Note, however, that establishing an ITA is only a necessary but not sufficient condition for implicit recognition as it is only the empirical basis for further inferences. Establishing an ITA is the first step to inferring implicit recognition. In the second step, additional theoretical assumptions are necessary to draw such conclusions. For example, among other matters of validity, one needs to assume the measure of explicit recognition to be exhaustive (Reingold & Merikle, 1988; Schmidt & Vorberg, 2006; Schmidt, 2007, 2015). That is, participants’ explicit recognition performance should not be hampered by low motivation or other issues.

To conduct the sensitivity comparison, we first need the participants’ sensitivity in the explicit recognition task. This is already routinely computed from participants’ hit rates (repeated configurations correctly recognized as repeated) and false alarm rates (new configurations mistaken as repeated):

1 $$\begin{aligned} d'_\text {direct} = \Phi ^{-1}(\text {HR}) - \Phi ^{-1}(\text {FA}) \text {.} \end{aligned}$$

$\Phi ^{-1}$ is the inverse cumulative normal density function. We label this sensitivity “direct” because it is a direct measure of recognition performance from explicit reports.

Next, we need an estimate for the sensitivity underlying the search task data. Because the RT data of the search task is typically taken as the basis for inferences about implicit recognition, we consider an ideal observer analysis on this data. Specifically, we assume participants had full explicit knowledge of their RT data making optimal use of the RT response in each trial to predict whether the stimulus configuration in this trial was repeated or new. From these predictions, we compute the sensitivity an ideal observer would have obtained in the explicit recognition task and compare it to participants’ actual explicit recognition sensitivity. If this ideal observer analysis does not produce an ITA then no real participant will and the RT data cannot be taken as evidence for the existence of some form of recognition (i.e., implicit recognition) that goes beyond explicit recognition.

With RT data being well approximated by log-normal distributions (Ulrich & Miller, 1993), the ideal observer would use a median-split classifier (see Supplement C in Meyen et al., 2022). The median split determines for each trial whether the RT response is relatively fast or relatively slow. Because the standard reasoning bases inferences on the result of faster mean RTs in repeated and slower mean RTs in new configurations, the ideal observer would classify (predict) that faster than median RT trials correspond to repeated configurations and slower than median RT trials to new configurations. Comparing these classifications against the true condition again yields hit and false alarm rates which can be evaluated in terms of sensitivity. Consequently, we use this ideal observer analysis to derive an estimate for the sensitivity underlying the search task:

2 $$\begin{aligned} d'_\text {indirect} = \Phi ^{-1}(\text {HR}) - \Phi ^{-1}(\text {FA}) \text {.} \end{aligned}$$

We label this sensitivity “indirect” because RT effects between repeated and new conditions are an indirect way of measuring recognition. The same analysis can be applied not only to RTs but also other indirect measures of recognition such as error rates, number of eye fixations, or any other trial-by-trial measure that indicates a difference between repeated vs. new configurations (e.g., see Schnepf et al., 2022, for an application to EEG data). Here, we will stick to the most prominent indirect measure: RTs. Computing the indirect (search task) sensitivity in this way simulates a situation in which participants have full explicit awareness of the RT data, which is supposedly produced by implicit recognition.

The sensitivity comparison then requires a test for a difference between the two sensitivities:

3 $$\begin{aligned} d'_\text {difference}&= d'_\text {indirect} - d'_\text {direct} \text {.} \end{aligned}$$

A difference in favor of the search task ($d'_\text {difference} > 0$) constitutes an ITA which can be evaluated, for example, by computing the mean sensitivity difference across participants and constructing a 95% confidence interval of the form 95% CI [*a*, *b*]. Only if 0 lies outside and below the interval, $0<a$, there is empirical evidence for an ITA. This, of course, corresponds to a classic significance test. Alternatively, Bayes factors (BFs) can be computed to evaluate this difference (Morey & Rouder, 2011; Rouder et al., 2009). In addition to confidence intervals, we will compute BFs using the standard implementation in the R package BayesFactor (Morey et al., 2015). We test the null hypothesis (sensitivity difference is zero) against the one-sided alternative hypothesis of an ITA (sensitivity difference is above zero). We use default priors of that package: a point null for the null hypothesis vs. a truncated Cauchy prior with scale $1/\sqrt{2}$ for the alternative hypothesis.

If this sensitivity comparison does not support an ITA, then participants could not have produced a higher sensitivity in the explicit recognition task even with full knowledge of their RT responses from the search task. Response in both tasks could therefore plausibly stem from one and the same source of recognition. Thus, without an ITA, inferring that search task RT responses indicate a second source of implicit recognition would be at odds with parsimony.

### Sensitivity comparison of studies based only on reported statistics

The sensitivity comparison we just outlined uses a median split and therefore requires full trial-by-trial data. For some studies, this full data is difficult to obtain or not available anymore (Wicherts et al., 2006). To nevertheless evaluate the evidence for an ITA in these studies, we present a method to estimate the indirect task sensitivity by only reported statistics from those studies. In particular, we will use the reported test statistic of a paired *t* test evaluating the RT effect between repeated and new configurations. From this *t* statistic, we will derive an unbiased estimate of the true search task sensitivity. As this method was derived in detail before (Meyen et al., 2022, Supplement D), we will only briefly sketch it here.

The true search task sensitivity—based on signal detection theory (Green & Swets, 1988)—is in our case the RT effect $\Delta$ divided by the trial-by-trial RT standard deviation $\sigma _\varepsilon$: $d'_\text {indirect, true} = \Delta /\sigma _\varepsilon$. We aim to estimate this sensitivity from an available *t* statistics which tests the RT effects against 0: $t = \hat{\Delta }/ SE$ where *SE* is the estimated standard error of individual RT effects. The *t* statistic is not straightforwardly converted to an estimate $d'_\text {indirect}$. While the numerator fits between $d'_\text {indirect, true}$ and *t*, the denominator does not. The standard error *SE* in the *t* statistic is not easily transferred into an estimate of the trial-by-trial standard deviation $\sigma _\varepsilon$ in $d'_\text {indirect, true}$. In particular, the standard error *SE* incorporates two sources of variance, the desired trial-by-trial variance, $\hat{\sigma }^2_\varepsilon$, but also the variance of RT effects between participants, $\hat{\sigma }^2_\text {effect}$. The latter represents how much the cueing effects vary from participant to participant. Following standard variance decomposition (e.g., see Rouder & Haaf, 2018), we formalize this relationship:

4 $$\begin{aligned} SE = \sqrt{ \frac{\hat{\sigma }^2_\text {effect} + \frac{4}{K} \hat{\sigma }^2_\varepsilon }{N} } \end{aligned}$$

where *N* is the number of participants and *K* is the total number of trials sampled per participant assuming balanced sampling so that each condition is sampled with *K*/2 trials per participant.

Relating *t* to $d'$ is therefore an underdetermined problem as a *t* value is generally consistent with a range of different $d'$ values. For a given *t* value, it is not clear to which degree *SE* originated from trial-by-trial variance ($\hat{\sigma }^2_\varepsilon$) vs. effect variance ($\hat{\sigma }^2_\text {effect}$). To solve this problem, we introduce a quantity, $q^2$, which determines the ratio between these two sources of variability:

$$\begin{aligned} q^2 = \frac{\sigma ^2_\text {effect}}{\sigma ^2_\varepsilon } \text {.} \end{aligned}$$

This variance ratio $q^2$ is related to the single-trial reliability of RTs. While typical reliability measures of RT effects depend on the number of trials (more trials reduce noise and increase the reliability of individual RT effects), our variance ratio $q^2$ considers the noise incurred in a single trial. By defining $q^2$ this way we can easily relate studies with different number of trials.

**Fig. 3:**
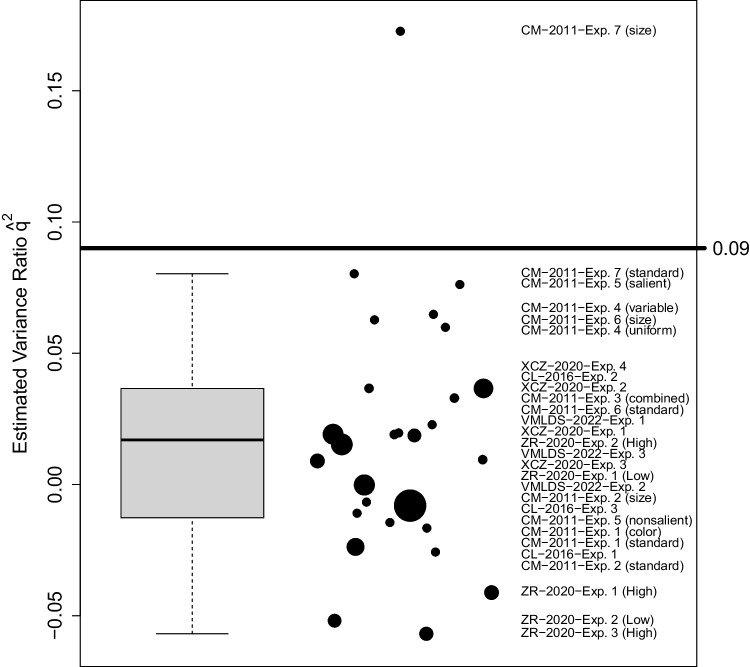
Variance Ratio $q^2$ Estimates from Trial-By-Trial Data. From the full trial-by-trial data of five studies we estimate $q^2 = \sigma ^2_\text {effect}/\sigma ^2_\varepsilon$. Except for a single outlier, all observed ratios fall below 0.09. Thus, we adopt $q^2 = 0.09$ as an assumption for our reanalyses of studies where full trial-by-trial data is missing. Using a high value like this increases the likelihood of confirming ITAs, see Section “Benefit-of-the-Doubt Approach”. Study labels are ordered according to their level of $q^2$. The diameter of the points is proportional to the log of the sample size

Knowing the variance ratio $q^2$ (and the number of participants *N* and trials *K*) of a reported *t* value solves the problem of underdetermination. With $q^2$, we can express the standard error from Eq. 4 as $SE = \hat{\sigma }_\varepsilon \sqrt{ \frac{q^2 + \frac{4}{K}}{N} }$, invert it, and obtain an estimate for the trial-by-trial standard deviation $\hat{\sigma }_\varepsilon = \sqrt{\frac{N}{q^2 + \frac{4}{K}}}SE$.

Then, based on the expected value of non-central *t* distributions (Hogben, Pinkham, & Wilk, 1961), we can derive an unbiased estimate for the underlying sensitivity:

5 $$\begin{aligned} d'_\text {indirect} = t\cdot c_{N,K,q^2}, \end{aligned}$$

where the constant $c_{N,K,q^2} = \sqrt{\frac{q^2 + \frac{4}{K}}{N}} \sqrt{\frac{2}{N - 1}} ~~\frac{\Gamma \left( \frac{N - 1}{2}\right) }{\Gamma \left( \frac{N - 2}{2} \right) }$ is chosen such that the expected value of our estimator $d'_\text {indirect}$ is the true underlying sensitivity, $E[d'_\text {indirect}] = d'_\text {indirect, true}$.

This reanalysis methods provides a way to estimate the search task sensitivity based only on a reported *t* value and knowledge of the variance ratio $q^2$. We provide an easy to access implementation on http://www.ecogsci.cs.uni-tuebingen.de/ITAcalculator/. For the following reanalyses, we will use the value $q^2 = 0.09$, which we will justify in the following section.

#### Estimating the variance ratio $q^2$

We will use the data from several studies where we do have access to the full trial-by-trial data to estimate $q^2$. More precisely, we will derive an estimator that will likely overestimate this variance ratio thereby overestimating $d'_\text {indirect}$ because $q^2$ appears in the constant of Eq. 5 in the numerator. Overestimating the search task sensitivity $d'_\text {indirect}$, in turn, increases the chances of confirming an ITA in our reanalysis. Conversely, this strengthens the conclusion of an absence of an ITA if none is found (see next section: “Benefit-of-the-Doubt Approach”).

To estimate $q^2$ from the trial-by-trial data, we must estimate two variances: $\hat{\sigma }^2_\varepsilon$ and $\hat{\sigma }^2_\text {effect}$. For the former, we compute the variances of RTs for each participant $\times$ condition (repeated/new) combination and pool those variance into a single estimate of the trial-by-trial variance, $\hat{\sigma }^2_\varepsilon$. Based on this and the observed standard error of RT effects, we invert Eq. 4 to obtain the estimated between-subject variance $\hat{\sigma }^2_\text {effect} = N\cdot SE^2 - \frac{4}{K} \hat{\sigma }^2_\varepsilon$. This effectively subtracts the amount of variability that would be expected purely based on trial-by-trial noise and leaves the variability that is due to underlying difference in true RT effects between participants. Thus, we obtain the variance ratio estimator:

6 $$\begin{aligned} \hat{q}^2 = \frac{N \cdot SE^2 - \frac{4}{K} \hat{\sigma }^2_\varepsilon }{\hat{\sigma }^2_\varepsilon } \text {~.} \end{aligned}$$

There were five studies from which we obtained full trial-by-trial data (Vadillo et al. , 2022; Xie, Chen, & Zang, 2020; Zhao & Ren, 2020; Conci & von Mühlenen, 2011; Colagiuri & Livesey, 2016). Each study contained multiple experiments and conditions in which we derived estimates for $q^2$ as shown in Fig. 3. Although $q^2$ is a ratio of variances and therefore must have a non-negative true value, some estimates of $q^2$ were negative. This happens when the standard error *SE* is lower than what would be expected based on the trial-by-trial noise alone ($\frac{4}{K} \hat{\sigma }^2_\varepsilon$, see Eq. 4). This can happen due to measurement noise when, by chance, participants’ RT effects are more similar than expected by chance. Allowing for negative values keeps the numerator of Eq. 6 unbiased.

Almost all estimated variance ratios fall below $\hat{q}^2 < 0.09$. Based on this, it is safe to assume that other studies—for which the trial-by-trial data is missing—will also have a smaller $q^2$. We will therefore assume $q^2 = 0.09$ for those other studies. Additionally, in Appendix A, we use different $q^2$ assumptions to calculate the main result to gauge the impact of this assumption. Results of the only outlier (Exp. 7 of Conci & von Mühlenen, 2011) are further addressed in the discussion.

**Fig. 4:**
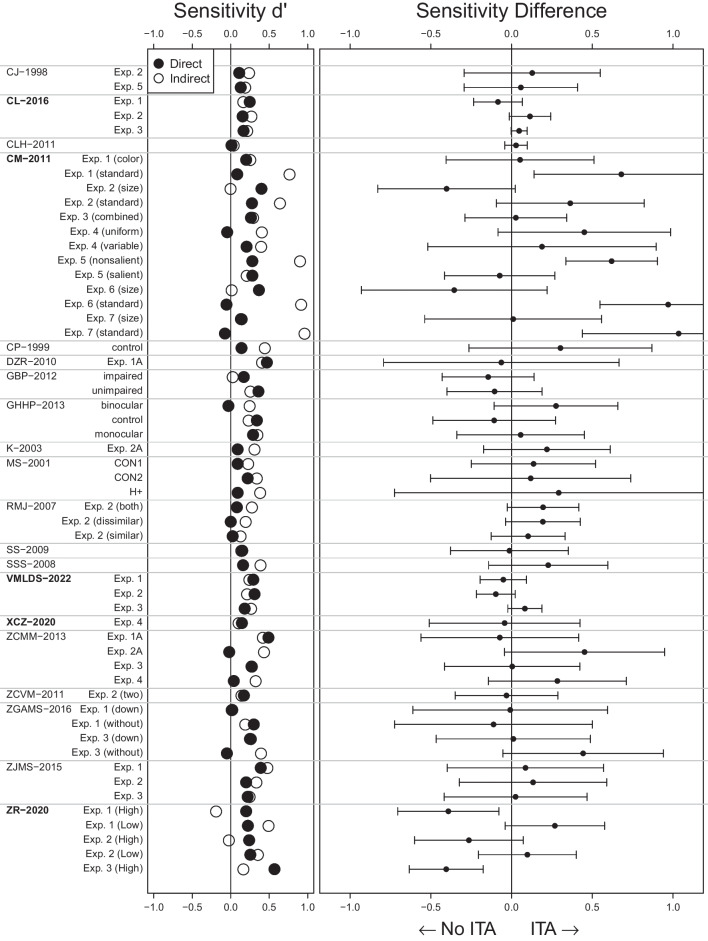
ITA Reanalysis Results. Sensitivities in 20 studies on contextual cueing. We plot sensitivities from the search task (indirect) and explicit recognition task (direct) on the left and their difference (indirect - direct) on the right. Error bars indicate 95% confidence intervals. Evidence for an ITA would be indicated by a confidence interval lying to the right of the 0 line without including the zero. All numerical values and label-study correspondence can be found in Supplement A. Studies for which we analyzed the full trial-by-trial data are marked in bold

At first glance, it is unclear whether $q^2 = 0.09$ is a plausible value. But a sanity check can easily be done by noting that the non-squared value *q* is the standard deviation of the true individual sensitivities (see Supplement D in Meyen et al., 2022). The assumption $q^2 = 0.09 = 0.3^2 \iff q = 0.3$ is equivalent to assuming that true individual search task sensitivities vary between participants at most with a standard deviation of 0.3. This is a reasonable assumption when the mean sensitivities are small with mean values around $d'_\text {indirect} = 0.3$ (see empty circles in the left subplot of Fig. 4).

Note that *q* refers to the standard deviation of *true* sensitivities; *q* is the standard deviation of $d'_\text {indirect}$ when infinitely many trials were sampled. Computing the standard deviation of observed search task sensitivities via the median split method will be larger due to additional measurement noise. Note also that we used a different (larger) value for $q^2$ in the reanalysis of priming studies (Meyen et al., 2022). Priming studies and contextual cueing studies differ in terms of their overall RT effects and also in the proportion of trial-by-trial noise vs. between-subject variances, such that adapting the variance ratio for the contextual cueing paradigm was necessary.

### Benefit-of-the-doubt approach

There are some choices made in this analysis method that need to be discussed. All of these choices are made in a way that gives the ITA the benefit of the doubt by increasing the estimated search task sensitivity, $d'_\text {indirect}$. In turn, our argument becomes stronger when we do not find an ITA although our analysis was biased in favor of finding it.

1. The contextual cueing effect on RTs unfolds over time. Although the absolute difference between repeated and new configurations often plateaus rapidly, the overall RTs also decrease throughout the course of the experiment. With faster overall RTs, the standard deviation of RTs reduces. Thus, the latest stages of an experiment should yield the largest RT differences, smallest standard deviation, and thus the largest sensitivities. Therefore, whenever possible, we reanalyze RTs from the last blocks of an experiment (typically labeled as the last epoch). Note that reducing the reanalysis to only the last trials of an experiment increases standard errors. But decreasing standard errors by taking more trials into account would also decrease estimated search task sensitivities $d'_\text {indirect}$ producing numerically less evidence for an ITA.
2. The median is computed for each participant individually. This allows for flexible classification adapted to each participant’s speed. Otherwise, a median computed over all participants would wash out the sensitivity. For example, consider a participant with very fast overall RTs. Their RTs in the new condition would be relatively slow when compared to only their median RT and, therefore, be correctly classified as coming from the new condition. But when compared to the median RT over all participants, their RTs would still be relatively fast and, therefore, incorrectly classified as coming from the repeated condition. In this way, using the median RT over all participants would lead to more incorrect classifications. Adapting the classification criterion to the individual participants gives the ITA an advantage by avoiding an underestimation of the search task sensitivity.
3. The median split can be proven to be optimal for log-normally distributed RTs (see Supplementary Material C in Meyen et al., 2022; results for ex-Gaussian instead of log-normal distributions will differ only marginally). The optimal classification approach maximizes the search task sensitivity in our reanalysis. More complex, nonlinear methods for classification are conceivable (Panis & Schmidt, 2016) but contextual cueing results are typically evaluated only based on a mean difference in RTs of repeated vs. new configurations. Thus, nonlinear methods would need further theoretical justification.
4. Taking the median over all observed RTs across blocks from a participant puts the indirect sensitivity estimate further into an advantage because it allows classifications based on a stable, optimal criterion computed from many blocks already for the first trials. In contrast, the explicit recognition task requires participants to set their internal criterion on the fly before the rest of the trials are presented in order to decide between recognized vs. new configurations. This makes the internal criterion for early trials less reliable and possibly reduces sensitivity in the explicit recognition task (Phillips, 2021). Thus, taking the median across all trials of many blocks of a participant in the search task—instead of a running median—favors finding an ITA.
5. As in the masked priming paradigm, some studies exclude participants based on a high explicit recognition task performance. This incurs the well known problem of regression to the mean (Barnett et al., 2004; Shanks , 2017): Assuming that participants are sampled in two tasks in which they exhibit the same sensitivity, excluding participants with a high sensitivity in one task biases results towards finding a sensitivity difference in favor of the other task. Except for one extreme case that we did not include in our reanalysis because 6 out of 16 participants (38%) were excluded (Zang et al., 2016, Experiment 2), we left these problematic results in our reanalysis potentially further compounding bias in favor of an ITA.
6. We chose a conservative estimate for $q^2$, as described in the previous section. This way, we most likely overestimate the true $q^2$ in other studies. Since larger $q^2$ lead to larger estimates of the search task sensitivity $d'_\text {indirect}$, we also overestimate $d'_\text {indirect}$ and, thereby, overestimate the evidence for an ITA.

With these methodological choices, the search task sensitivity $d'_\text {indirect}$ is likely to be overestimated. This makes it more likely to produce empirical evidence in favor of an ITA. With that, we make our critique of ITAs conservative.

## Study selection

We reanalyzed a total of 20 studies. At first and to establish whether there is a problem in the contextual cueing literature, we exploratively reanalyzed five studies that were fundamental to the field (e.g., Chun & Jiang, 1998) and easily accessible (e.g., Zhao & Ren, 2020), see Supplement A for details. We then chose to reanalyze a set of studies that was previously considered in the reanalysis of Vadillo et al. (2016). To avoid well-known problems of motivated data analysis (Kerr , 1998; Nosek et al., 2018; Simmons et al., 2011; Wagenmakers et al., 2012), we preregistered this part of the reanalysis (https://aspredicted.org/rw6tt.pdf) including those studies that either reported the statistics necessary for our reanalysis or provided their full trial-by-trial data.

With that, we reanalyzed 12 more studies based on their reported statistics and three more studies based on their full data, which we received after contacting the corresponding authors. We had contacted nine corresponding authors but only these three responses were positive. Taken together, we were able to reanalyze 15 more studies from our preregistration. We did not reanalyze any additional studies. All details on the studies as well as the reanalysis results can be found in Supplement A.

## Sensitivity comparison results

We illustrate our results by first reanalyzing one particular study as an example. Then, we show results for all reanalyzed studies in our main Fig. 4. Details of all reanalyzed studies can be found in Supplement A.

### Example reanalysis based on reported statistics

For our example reanalysis, we focus on Experiment 2 of the seminal study by Chun and Jiang (1998). The other experiments in the study did not feature a direct recognition task (Experiments 1, 3, 4, and 6) or produced analogous results (Experiment 5). In Experiment 2, $N=14$ participants conducted $K_\text {indirect} = 96$ trials in each of the six epochs of the search task. Afterwards, each participant conducted $K_\text {direct} = 24$ trials in the explicit recognition task.

Chun and Jiang (1998) found the typical pattern of results: A clear RT effect in the search task and a close to chance sensitivity in the explicit recognition task. In the last epoch of the search task, participants responded 50 ms faster to repeated than new configurations, $t(13) = 2.61$ (their Table 2 on p. 42). The explicit recognition task sensitivity was $d'_\text {direct} = 0.11$ which we computed from the reported hit rate $HR = 39.3\%$ and false alarm rate $FA = 35.1\%$. Following the sensitivity comparison approach, we estimate the search task sensitivity from the *t* value by computing constant $c_{N = 14,K = 96,q^2 = 0.09} = 0.091$ and multiplying it to the *t* value so that we get $d'_\text {indirect} = t \cdot c_{N = 14,K = 96,q^2 = 0.09} = 2.61 \cdot 0.091 = 0.24$ (corresponding to the empty circle in the first row of Fig. 4).

There is a numerical difference between these two sensitivities hinting towards an ITA, $d'_\text {difference} = d'_\text {indirect} - d'_\text {direct} = 0.24 - 0.11 = 0.13$ (point in the first row of the right subplot in Fig. 4). But taking measurement error into account (see Supplement D in Meyen et al., 2022, for the derivation), we find a large standard error $SE_\text {difference} = 0.20$ such that the numerical difference can plausibly stem from measurement error, $t(13) = 0.66$, $p =.52$, 95% CI $[-0.29, 0.55]$ (this confidence interval corresponds to the error bars in the first row in the right subplot in Fig. 4). Bayes factor analysis tends to favor the null hypothesis of no ITA, $BF_{10} = 0.47$ (first row in Fig. 6).

By any standard, this numerical difference is not to be considered as a conclusive demonstration of higher sensitivity in the search task as compared with the explicit recognition task. But this is the strongest result from the original study! Reanalyzing any other epoch from Experiment 2 or the results from Experiment 5 leads to even weaker evidence for an ITA. Even under the favoring conditions of our benefit-of-the-doubt approach, the seminal study by Chun and Jiang (1998) presents only weak to no evidence for an ITA.

### Reanalysis of 20 contextual cueing studies

Finally, we applied our reanalysis to a total of 20 studies with 56 conditions and experiments. Our aim was to gauge how much evidence for an ITA there is in each study. Many but not all of these studies (15 out of 20) inferred that contextual cueing was implicit. Some of these studies additionally use correlation-based arguments, which we will address in the next section. For now, we want to determine whether there is evidence for ITAs.

**Fig. 5:**
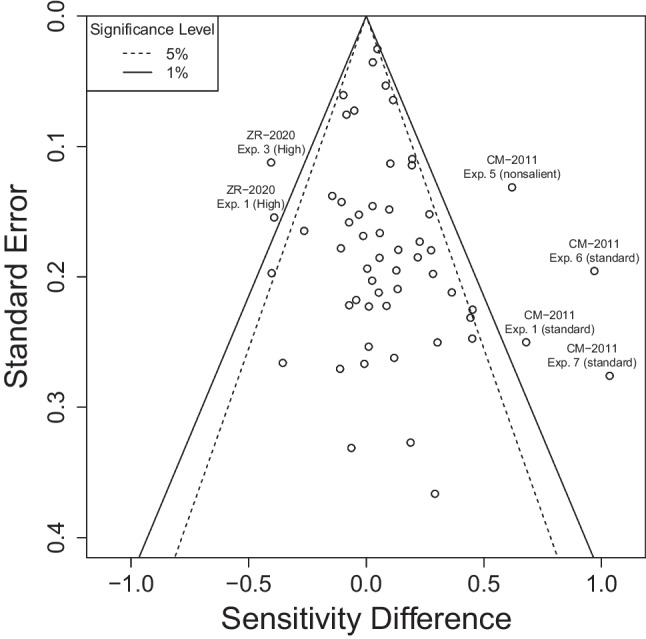
Funnel Plot of ITA Reanalysis Results. The funnel plot shows the sensitivity differences ($d'_\text {difference} = d'_\text {indirect} - d'_\text {direct}$) in relation to their standard errors. Each data point corresponds to one experiment or condition from the reanalyzed studies. More precisely measured sensitivity differences (smaller SEs, towards the top) converge closer to 0. Dotted and solid lines show significant deviations from 0 for significance levels of 5% and 1%, respectively

Figure 4 shows our main result: For each experiment and condition in the reanalyzed studies, we show in the left subplot sensitivity estimates for the explicit recognition task (direct, filled circles) and the search task task (indirect, empty circles). Already here it is apparent that, in many cases, the search task sensitivity is even numerically smaller than the explicit recognition task sensitivity. In the right subplot we show the sensitivity differences (indirect - direct) together with their 95% confidence intervals. Almost all of these confidence intervals include the vertical 0 line and many scatter to the left.

No study shows consistent evidence for an ITA throughout its experiments and conditions. In fact, the majority of the studies did not provide evidence for an ITA in any of their experiments or conditions (19 out of 20 studies). There was only one from the 20 reanalyzed studies for which we found evidence for an ITA in half of their conditions (Conci & von Mühlenen, 2011, labeled CM-2011). The same experiments had another condition in which there was no ITA present. Nevertheless the study does not differentiate between these conditions (“no explicit awareness of the display repetitions”, p. 225). In contrast, another study (Zhao & Ren, 2020, labeled ZR-2020) shows the opposite result of an ITA in two conditions: a clearly higher sensitivity in the explicit recognition task than in the search task.

Even without taking measurement errors into account, only 37 of the 56 reanalyzed experiments and conditions (66%) showed a numerical difference of sensitivities in favor of an ITA—which has to be seen in light of our benefit-of-the-doubt approach that tends to overestimate evidence for an ITA.

To further judge the evidence for ITAs, we show the same data in Fig. 5 as a funnel plot (Sterne et al., 2011). The funnel plot relates the observed sensitivity differences (x-axis) to their standard error (y-axis). More precise measurements of the sensitivity differences (smaller SE, upwards in the plot) lead to mean differences converging to 0 (where the funnel is centered at)—indicating no ITA.

**Fig. 6:**
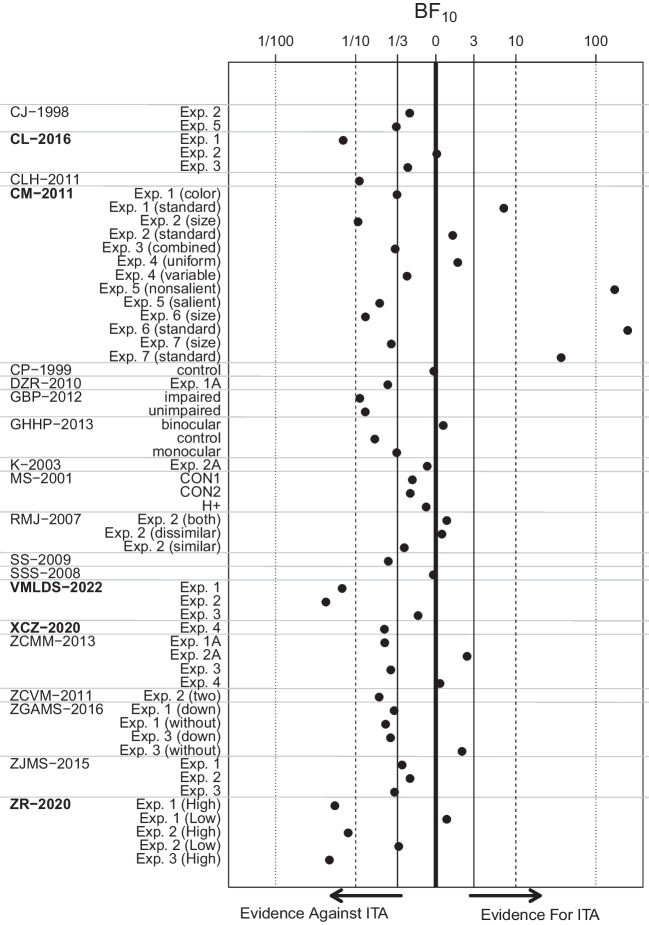
Bayes Factors Suggest Evidence Against ITAs. Bayes factors (BFs) for the sensitivity differences as shown in Fig. 4. BFs larger than 3 indicate evidence for an ITA, BFs smaller than 1/3 indicate evidence against an ITA, and BF between 1/3 and 3 are inconclusive. Mostly, BFs point towards evidence for no ITA. Studies for which we analyzed the full trial-by-trial data are marked in bold. For a further, in-depth discussion of Bayes factors with respect to the standard reasoning, see also Appendix B. BFs are drawn to log scale

So far, we have considered the *absence of evidence* for ITAs. Because providing *evidence for the absence* of an ITA is less straight-forward in Frequentist statistics, we additionally provide a Bayesian reanalysis. Figure 6 shows the Bayes factors when testing the sensitivity difference against zero. We found 28 out of 56 conditions and experiments provide evidence against the existence of an ITA ($BF_{10} < 1/3$). Another 24 conditions and experiments were inconclusive ($1/3< BF_{10} < 3$). Only the previously mentioned study (Conci & von Mühlenen, 2011, labeled CM-2011) provided four conditions with more than anecdotal evidence for ITAs ($BF_{10} > 3$). That is, the Bayesian analysis supports evidence *against* ITAs in the majority of studies. These results are consistent with the Frequentist analysis from above further supporting the overall absence of evidence for an ITA.

### Validation of reanalysis method based on reported statistics

**Fig. 7:**
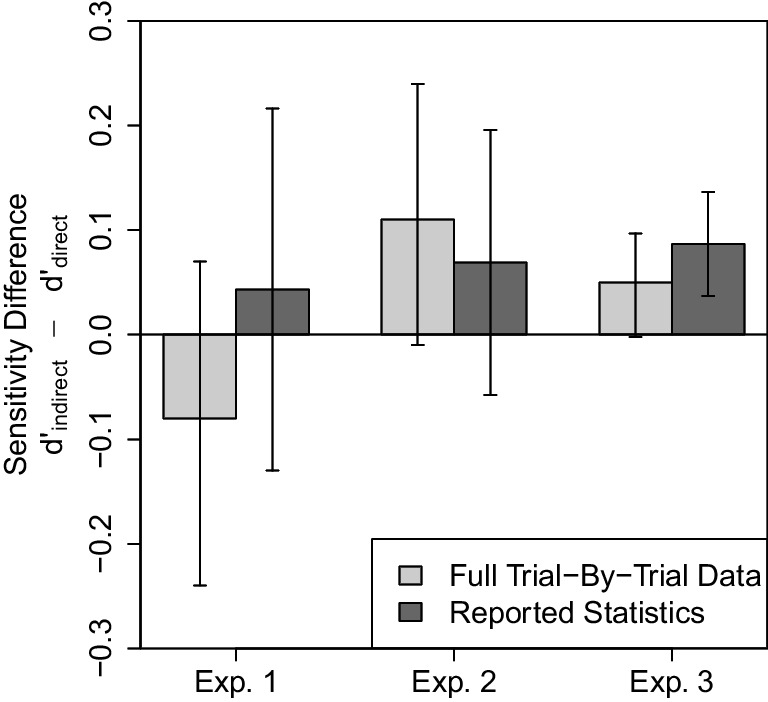
Comparison of Reanalysis Results Based on Full Trial-By-Trial Data vs. Only Reported Statistics in Colagiuri and Livesey (2016). Light grey bars show results of the sensitivity comparison based on the full data (more accurate) while dark grey bars show results based on the reported statistics (using the assumption $q^2 = 0.09$). In Experiment 1 and 3, the estimate based on reported statistics goes in favor of an ITA (sensitivity difference $d'_\text {difference}$ is overestimated). This will happen in most cases. In Experiment 2, the reported statistics underestimate the sensitivity difference, which can happen in few cases due to measurement error. Error bars indicate 95% confidence intervals

Now we demonstrate that our results based on the reanalysis from reported statistics matches that from the full trial-by-trial data. For this validation, we conducted both reanalyses on the data from Colagiuri and Livesey (2016) which the authors shared with us. The results are displayed in Fig. 7. Except for small numerical differences both reanalyses closely match.

### Interim summary

Taken together, the evidence for ITAs is weak to non-existent. RTs in the search task seem to be, for the overwhelming majority, not more sensitive than participants’ explicit reports. No study presents consistent evidence for an ITA throughout their experiments and conditions (Fig. 4). The more precisely a sensitivity difference is measured, the more it converges to 0 (Fig. 5). Bayesian statistics tend to favor the null hypothesis of no difference in the majority of the cases (Fig. 6). In the context of our benefit-of-the-doubt approach in which we set up our analysis to favor finding an ITA, these results make it very plausible that the sensitivity underlying search task RTs is not larger than the explicit recognition task sensitivity. In short, there seems to be no consistent evidence for ITAs.

These analyses so far were done on an aggregate level across all participants. Next, we turn to an individual ITA analysis. This individual-level analysis is becoming theoretically more relevant as it relates to recent correlation-based arguments for implicit recognition. We will first explain this reasoning, point out two of its issues, and then show how our sensitivity analysis can be used to evaluate these correlation-based arguments as well.

## Correlation-based arguments to infer implicit recognition

We now turn to arguments that are based on the empirical observation that explicit recognition sensitivity and search task RT effects show only a low correlation. The intuitive appeal of this reasoning: When search task RT effects are practically independent of explicit recognition, there must be a driver of search task effects that is independent of explicit recognition, which is then thought to be *implicit* recognition.

A very strong empirical demonstration of a low correlation is presented by Colagiuri and Livesey (2016). In a commendable sample of $N=766$ (Experiment 3), Colagiuri and Livesey found a correlation as low as $r =.03$ from which they inferred that the search task RT effects are independent of explicit recognition. Other studies find the same result and have argued in a similar manner (e.g., Geyer et al., 2010; Conci & von Mühlenen, 2011; Geringswald et al., 2012; Geringswald et al., 2013; Zang et al., 2015; Chun & Jiang, 1998; Chun & Phelps, 1999).

### The problems of interpreting low correlations as implicit recognition

First, it is important to note that observing a low correlation between search task RT effects and explicit recognition sensitivity does not immediately show that the underlying constructs are independent or that there are two distinct constructs (i.e., an explicit and an implicit form of recognition). Vadillo et al. (2022) have discussed the issue of reliability: Search task RT effects incorporate a large degree of noise (e.g., motor noise) and the explicit recognition task features typically only very few trials per participant. This makes both measures inherently unreliable. But unreliable measures do not even correlate highly with themselves. The same participant, when measured again, can display a substantially different RT effect or a different explicit recognition sensitivity due to this measurement unreliability. Therefore, one cannot expect a high correlation between search task RT effects and explicit recognition performance in the first place. When correcting for the low reliability of these measures, Vadillo et al. showed that no strong evidence for implicit recognition emerges from the current data (see also Malejka et al., 2021).

Also, there is evidence that a positive correlation exists (Annac et al., 2019; Geyer et al., 2020) but, often, small correlations are overlooked. For example, consider that Schankin et al. (2008) found $r=.29$ in a sample of $N=16$ participants and inferred that there is no relationship between explicit recognition and search task RT effects (see similar results in Rosero et al., 2019, and Zellin et al., 2013). But the standard error of a correlation in a sample of $N=16$ participants can be up to approximately $SE = 0.28$ and the 95% confidence interval would be consistent with rather high correlations, $95\%\text {~CI~}[-0.24, 0.69]$ (Fisher, 1948). Clearly, this result is not conclusive evidence for the absence of a small correlation, that can be substantial after accounting for measurement error.

Second, caution is necessary when interpreting low correlations as evidence for implicit recognition even after reliability issues are taken into account. Loosely related measures do not necessarily imply loosely related underlying constructs due to validity issues. We want to draw attention to two such major issues here. Both issues substantially impact the interpretations of low correlations; both raise doubt about whether a low correlation can be a valid basis for inferring implicit recognition.

**Fig. 8:**
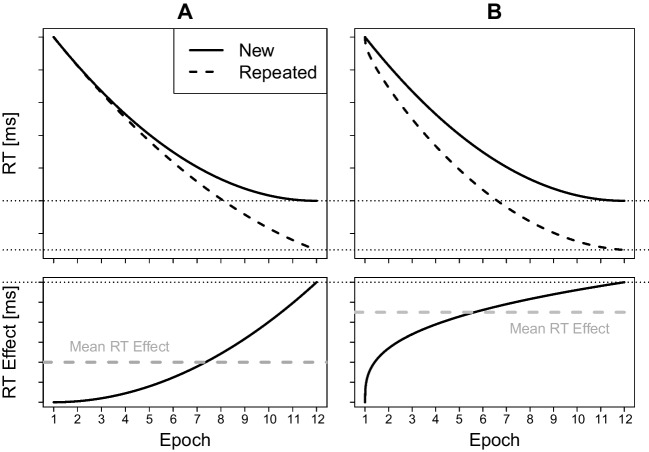
Variability in learning rate affects mean RT effect. Hypothetical data of two participants, A and B. Both participants reach the same contextual cueing RT effect in Epoch 12 as indicated by the dotted lines. However, A’s RT effect increases later than B’s. Thus, the mean effects averaged across epochs differ: A has a lower mean RT effect than B indicated by the grey dashed lines. Assuming that the RT effect at the end of the experiment (Epoch 12) corresponds to a participant’s recognition, the variability in the speed of RT effect emergence induces variability in the mean RT effects which in turn diminishes the correlation between recognition and mean RT effects

#### Variability in learning rates diminishes correlations

Measures such as RT effects are imperfect in capturing the target construct, implicit recognition, because they can be confounded by other constructs. Consider again the study by Colagiuri and Livesey (2016). They measured search task RT effects as an average RT effect across all blocks throughout their whole experiment. Including all blocks introduces a confound: Their RT effects do not only incorporate recognition of the repeated configurations but also learning rates. See Fig. 8 for an illustration. Suppose participants vary in their learning rates, that is, some develop an RT effect slowly (Participant A in the figure) while others develop it fast (Participant B). Variability in learning rates have been demonstrated for different presentation conditions (Tseng et al., 2011) so it is plausible that they vary between participants as well—some may learn faster than others. Then, even if two participants reach the same RT effect at the end of the experiment (corresponding to the same level of recognition), their mean RT effects will vary.

This variability introduced by different learning rates diminishes the correlation between RT effects and recognition. But then, a loose correlation is not clear evidence for independent sources of recognition; it can plausibly (in parts) stem from learning rate variability.

#### Nonlinearity and nonmonotonicity diminishes correlations

A nonlinear functional relationship between the two measures can additionally reduce correlations even if both measures are entirely derived from the same source. And the relation between search task RT effects and explicit recognition is almost certainly nonlinear: One would not expect RT effects to continue increasing as explicit recognition sensitivities increase. Instead, RT effects will plateau inducing a nonlinear relationship. Such nonlinearities are demonstrated in priming, for example, by Zerweck et al. (2021) and Kinder and Shanks (2003), who even show RT effects and direct measures to be related by an inverse U-shape! In contextual cueing, Colagiuri and Livesey (2016) even suggest a mechanism for this (p. 2008): “Another possibility is that recognition of a repeated pattern during the visual search task slows down performance because additional mental processes are engaged.”

However, the current debate in contextual cueing and other paradigms (Berry et al., 2012; Colagiuri & Livesey, 2016; Malejka et al. , 2021; Vadillo et al. , 2016, 2022) is based on a linear single-source model (Berry et al., 2006; Berry et al., 2008a; Berry et al., 2006; Berry et al., 2008b) and computes correlations, a measure of linear relationship strength. If the data does not seem to support such a linear relationship, it is indeed *one* possibility that search task RT effects and explicit recognition sensitivities are independent—which would lend credibility to the notion of implicit recognition. But it is *another* possibility that the RT effects are derived from explicit recognition in a nonlinear manner. To conclusively infer the former, the latter must be ruled out. Convincing evidence for this is still lacking in the contextual cueing paradigm.

### Sensitivity comparison to the rescue: Replace correlations by individual ITAs

One solution to the quandary of interpreting correlations as evidence for independent processes is, again, the sensitivity comparison. The idea is to test for ITAs but now on the individual level to answer the question: Are search task sensitivities higher than explicit recognition task sensitivities *for individual participants*? If empirical ITAs could be demonstrated for a sizeable proportion of participants, then the empirical first step for inferring implicit recognition is established. A second interpretation step is then required in which additional assumptions need to be made and issues of validity need to be discussed.

With no ITAs at the individual level, even a very low or zero correlation can not definitively rule out the possibility of RT effects being fully driven by explicit recognition. It is possible that each individual participant’s RT effect is entirely derived from their explicit recognition task sensitivity but that the above mentioned problems nevertheless produce a low correlation.

Thus, we evaluated evidence for individual ITAs in the data provided by Colagiuri and Livesey (2016). Because their Experiment 3 has such an exceptionally large sample size ($N = 766$), the proportion of individual participants demonstrating an ITA can be investigated with sufficient precision. Since each participant performed multiple trials per task, we used a bootstrap method to estimate standard errors for each participant. We then performed one-sided *t* tests with $\alpha = 5\%$ comparing each participants’ individual sensitivity difference against 0. Results of this analysis showed that only 6.3% (95% CI [4.7, 8.2]) of the participants (48 out of 766 participants) showed a significant ITA. This proportion lies well within the range of false positives ($\alpha = 5\%$) that we would expect if no participant had a true ITA.

To summarize, there is no evidence for individual ITAs in this experiment. Despite the low correlation between RT effects and explicit recognition sensitivity, we did not find evidence for the existence of participants with search task sensitivity beyond what they can explicitly report.

## Discussion

We have shown that the standard reasoning to infer unconscious processing in the contextual cueing paradigm is flawed. In the same way in which it has produced misleading interpretations in masked priming studies, it also led to problematic interpretations in the contextual cueing paradigm where ITAs are frequently assumed for making inferences about implicit recognition. Under scrutiny, almost no ITAs can be found in these studies: The appropriate sensitivity comparison almost never indicates that RTs are (up to measurement error) more sensitive than participants’ explicit reports. In short, there is no consistent evidence for ITAs. But without ITAs, a simple and parsimonious interpretation of the current data is that the same source of weak recognition drives participants’ responses in both, the explicit recognition task and the search task. Of course, it is still possible that a second source of implicit recognition drives contextual cueing effects but more convincing results have to be produced to make such a strong claim. As it stands, not much has changed in the past decade (Berry et al., 2010, see also Eriksen , 1960): Genuine evidence for implicit recognition beyond what participants can explicitly report is—in almost all cases—still lacking.

In our reanalysis, we found four (out of 56) results in which an ITA emerged and all these results came from a single study (Conci & von Mühlenen, 2011). But as we noted earlier, finding an ITA is only the first step in inferring implicit recognition. Other issues of validity must be considered. Here, one issue is that all four of those conditions showed large RT effects (151-$211~\text {ms}$, $p\text {s} <.014$) between repeated and new configurations in the very first block already. That is, even before the repeated displays were shown a second time, responses to them were faster. These RT effects in the first block cannot be due to recognition: Recognition can only occur when the displays are repeated for the first time in block two. Thus, RT effects likely stem from a source other than recognition, for example, from the repeated displays being initially easier to solve than the new displays. Therefore, the ITAs in these four conditions do not provide clear evidence for implicit recognition either.

We have additionally shown how correlation-based arguments of inferring two independent sources of recognition, one explicit and one implicit, can also go wrong. There are multiple factors that can diminish correlations even when there is only one source of recognition. For now, one source of recognition suffices to explain the current data in the search task, which should be the default hypothesis (see Phillips , 2021, for this general argument). The onus is on the proponents of implicit recognition to present strong evidence ruling out this plausible interpretation.

### Should results be aggregated into a meta-analysis?

In our reanalysis we have abstained from conducting a meta-analysis. Aggregating over the studies would not be appropriate because they vastly differ in many regards. Some studies use different population such as patients with amnesia (Chun & Phelps, 1999), patients with brain lesions (Manns & Squire, 2001), or children (Dixon et al., 2010). Others drastically vary the experimental design in which they present the stimulus displays, for example, with a simulated visual scotoma (Geringswald et al., 2012). Aggregating results across these studies would not appropriately account for their divergent research questions.

More importantly, due to our benefit-of-the-doubt approach, a meta-analysis would accumulate the bias in favor of an ITA that we introduced with our benevolent reanalysis strategy. In contrast, our goal here was to evaluate the evidence for ITAs in each of the studies individually. A meta-analysis should not be necessary because each study claiming implicit recognition based on their results should provide self-contained evidence for an ITA. But note that not all studies inferred contextual cueing to be implicit, see Supplement A for details on each study’s interpretations. Therefore, we abstain from reporting aggregated statistics such as averaging sensitivity differences or multiplying Bayes factors across studies.

Another avenue of research is to investigate ITAs on a group level. It is already known that there are group differences between, for example, young and old participants with regard to cueing effects (Howard et al., 2004). There are also differences between healthy participants and memory-impaired patients in some (Manns & Squire, 2001) but not all studies (Chun & Phelps, 1999; Barnes et al., 2010). Building on these already known difference in contextual cueing effects has the potential of identifying ITAs in specific subgroups. Yet another approach is to establish double dissociations (Schmidt & Vorberg, 2006; Schmidt, 2007; Schmidt & Biafora, 2022): By demonstrating that explicit recognition sensitivity and RT effects can be independently manipulated, evidence for implicit recognition may be established (e.g., Biafora & Schmidt, 2020 show a dissociation in priming). However, for contextual cueing, this evidence is still lacking.

### Converging evidence from non-binary explicit recognition tasks

In line with our results, other studies using non-binary explicit recognition tasks have also produced evidence questioning the implicit nature of contextual cueing (Annac et al., 2019; Kroell et al., 2019; Geyer et al., 2020). These studies have used more refined recognition tasks: They employed target generation tasks in which participants see a display where the target T is replaced by a distractor L and have to guess where the target T was in the original display (Jiang & Chun, 2003). Because such tasks better capture participants’ explicit recognition capabilities, these studies usually show above chance recognition performance as well as sizeable correlations with the search task RT effects. Moreover, Annac et al. (2019) computed *z*-scores in the explicit recognition and the search task. They also found no difference between the tasks. But note that *z*-scores are affected by the number of trials employed in a task. This limits the interpretability of their result while our sensitivity comparison does not suffer from this problem. While sampling more trials leads to an increase in *z*-scores, sensitivities are only measured more precisely and their mean value does not change. Nevertheless, these findings - combined with our results - make it even more plausible that contextual cueing is driven by (a small amount of) explicit recognition.

As of now, our sensitivity comparison can only be applied to binary explicit recognition tasks, which is why we did not reanalyze studies using quadrant generation tasks here. Future work has to relate RT effects to the explicit recognition performance in these more complex tasks.

### Restricting the sensitivity comparison to learned displays

A suggestion during the review process was to apply the sensitivity comparison on only a subset of learned displays because it seems that contextual cueing RT effects stem from only a few of the repeated displays that are learned while the other displays are not learned. Previous studies identified which displays were learned based on comparably large RT effects or, alternatively, a high explicit recognition task performance for particular repeated displays (Geyer et al., 2010, 2020; Smyth & Shanks, 2008). They typically find that only 2-4 out of 8 repeated displays are learned. However, conducting further analyses–such as our sensitivity comparison–on the displays selected this way creates a problem of regression to the mean (Shanks, 2017): For example, when first selecting repeated displays based large reaction time effects and then comparing the underlying sensitivity, it is to be expected that a sensitivity difference appears simply due to the selection procedure and even if there is no underlying sensitivity difference. We nevertheless present these analyses in Supplement B but caution to interpret the results. Future research may provide appropriate methods to identify learned displays without this methodological problem (e.g., by deliberately manipulating the displays as in Geyer et al. , 2020) and demonstrate convincing ITAs.

Note that the variance ratio parameter $q^2$ is still meaningful even if there was an underlying dichotomy of learned vs. not learned displays. Whether a unimodal (all displays learned relatively equally) or a bimodal distribution (learned vs. not learned displays) underlies each participant’s mean effect does not change how the between-subject variance $\sigma ^2_\text {effect}$ is to be estimated. Therefore $q^2$ does not change under this consideration. It is conceivable that future research isolating learned displays can produce situations with larger $q^2$ and consistently find ITAs. However, this is not the case in the reanalyzed studies.

### Implications for interpretations about implicit recognition

Some of the studies’ theoretical discussions must be revised under the new light of missing ITAs. For example, Tseng et al. (2011) have investigated similarities between implicit and explicit learning in the contextual cueing paradigm finding surprising similarities. Given the lack of evidence for ITAs, one would not distinguish between implicit vs. explicit learning in the first place but rather consider only one source of learning. Then, the observed similarities do not come as a surprise anymore. In another example, Chun and Jiang (2003) remark that implicit learning can be long-lasting. This could be considered surprising as implicit processes are assumed to be short-lived but, if cueing effects stem from the same source as explicit recognition responses, this again is not surprising anymore. Kunar et al. (2006) observe that explicit instructions can influence contextual cueing which, again, is well within expectation if cueing is not assumed to be driven by a separate, implicit source of recognition to begin with.

We want to stress that each study makes valuable contributions in their own right. Many claims in those studies did not pertain to the question of whether or not cueing was implicit. By refuting current evidence for ITAs, we do not want to diminish these contributions. Independent of the interpretations regarding the implicit nature of contextual cueing, there is a plethora of relevant and valid findings in these studies. We only question whether these findings should be interpreted as implicit phenomena.

### Limitations and strengths of the sensitivity comparison

One weakness of our reanalysis method based on only the reported statistics is that it incurs more measurement error than an analysis based on the full trial-by-trial data. A trial-by-trial reanalysis of all studies in the contextual cueing paradigm would be beyond the scope of this article. Future efforts in the field are necessary to scrutinize the evidence using trial-by-trial data. This will make measures of sensitivity differences more precise (smaller error bars in Fig. 4). But note that we used a liberal assumption on $q^2$ so that, when replacing this assumption by the trial-by-trial data, not only precision but, likely, also numerical differences in favor of an ITA will vanish.

Another concern with the sensitivity comparison is that RTs in the search task are corrupted by neuro-muscular noise. This reduces the observed sensitivity in this task. The underlying sensitivity (before noise in the translation from recognition to RT) may have been higher. With that, an ITA may exist after accounting for this additional noise. But it is on the proponents of implicit recognition to present evidence for this. The strong claim about implicit recognition cannot be based on the mere assumption of an ITA in some underlying measure—it has to be empirically demonstrated. Moreover, it is possible that responses in the explicit recognition task are also corrupted by additional noise in the translation from recognition to an explicit response: When recognition is weak, binary decisions are to a large degree arbitrary and arbitrary decisions suffer from a similar kind of noise as well (as, for example, demonstrated for readiness potentials by Maoz et al., 2019, and Schurger et al., 2012).

The sensitivity analysis allows researchers to go beyond the outdated significant/non-significant distinction in the two tasks and move towards what has been labeled the “new statistics” (Cumming , 2013, 2014; Cumming & Calin-Jageman, 2016; Kruschke & Liddell, 2018; Wasserstein, Schirm, & Lazar, 2019) by focusing on an estimation problem rather than a decision problem. The standard reasoning looks at the two tasks, search task and explicit recognition task, in separation and attempts to make decisions regarding two questions: “Is there a contextual cueing effect?” and “Is there explicit recognition present?” Usually, the answers are yes, there is a contextual cueing effect, and no, there is no explicit recognition. But this reasoning is erroneous as we have shown. Notably, this is not only a problem of Frequentist statistics; Bayesian statistics suffer from equivalent problems albeit less severe (Sand & Nilsson, 2016; Dienes & Overgaard, 2015)–see Appendix B for a demonstration of the problems in Bayesian statistics. The solution is not to replace one school of statistics with another but rather to apply the appropriate comparative test in either of them. Here, the sensitivity comparison replaces the two decision problems and puts in their place an estimation problem: How large is the difference in sensitivities between explicit recognition and search task RT effects? How much recognition is there beyond what participants can explicitly report? Measuring this sensitivity difference allows the field to progress into a more empirical direction.

**Fig. 9:**
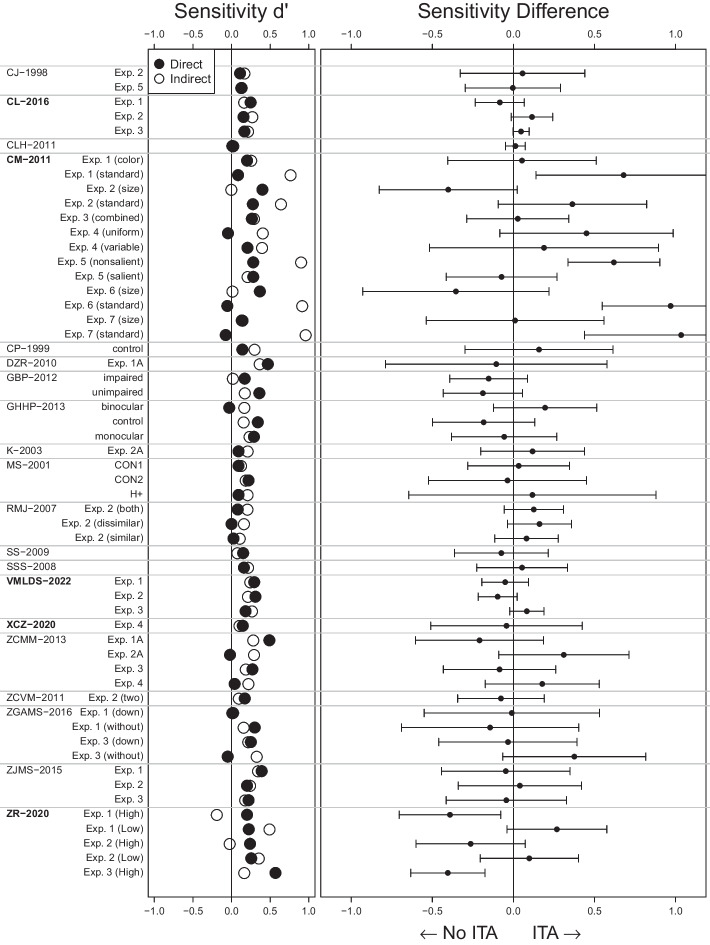
ITA Reanalysis Results. Similar to Fig. 4 but with $q^2 = 0.0225 = 0.15^2$ (instead of $q^2 = 0.09 = 0.3^2$). This lower $q^2$ value attributes more variance to the trial-by-trial noise and therefore produces smaller reanalyzed sensitivity in the search task (indirect task). Results under this plausible assumption produces less evidence for ITAs

**Fig. 10:**
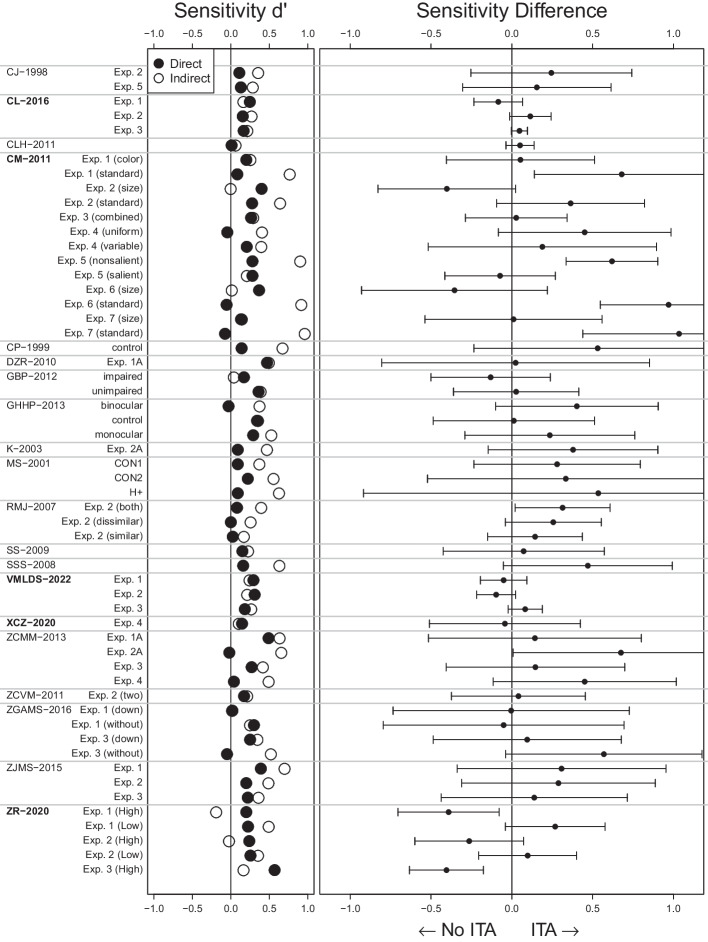
ITA Reanalysis Results. Similar to Fig. 4 but with $q^2 = 0.25 = 0.5^2$ (instead of $q^2 = 0.09 = 0.3^2$). The larger $q^2$ value attributes less variance to the trial-by-trial noise and therefore produces larger reanalyzed sensitivity in the search task (indirect task). Results under this extreme assumption produce numerical results that point more towards ITAs. But since more variance is attributed to between-subject differences this way, standard errors are also larger

### Conclusion

Adopting the sensitivity comparison to demonstrate an ITA will help the contextual cueing paradigm to establish itself in the larger field of unconscious and implicit memory research. As of now, this field is plagued by doubts of not being rigorous enough (Michel et al., 2018). Without reliable inference methods, contextual cueing runs the risk of being one of the many paradigms promising but ultimately failing to provide convincing empirical evidence for implicit processes (Newell & Shanks, 2014).

### Supplementary Information

Below is the link to the electronic supplementary material.

Supplementary file 1 (pdf 332 KB)

## Data Availability

Not applicable.

## Appendix A

### Reanalysis with different $q^2$ values

The reanalysis we conducted is based on one key variable, $q^2$. We took great care in estimating this variable, taking multiple experiments into consideration and used an estimation that likely overestimated $q^2$ to not produce overly critical reanalysis results (see Fig. 3 in the main text and section “Benefit-of-the-Doubt Approach”). Nevertheless, one may ask how the analysis would have looked if we assumed a different $q^2$ value instead. In our main reanalysis, we used the value $q^2 = 0.09$. Here, we present our main results figure (Fig. 4) in two variants: One with a lower and more plausible $q^2 = 0.0225$ in Fig. 9 and one with a larger $q^2 = 0.25$ in Fig. 10. By and large, these analyses also do not provide convincing evidence for ITAs.

## Appendix B

### The flaw of the standard reasoning in frequentist and bayesian statistics

In the main text, we cautiously did not frame the fallacy of the standard reasoning in terms of null hypothesis significance testing. We did this because the fallacy is independent of the statistical framework—be it Frequentist or Bayesian—used to determine a clear effect in the search task and a low sensitivity in the explicit recognition task.

Previous studies have often fallen for the fallacy of the standard reasoning using Frequentist null hypothesis testing. They inferred a difference between the two tasks from a significant result in one task and a non-significant result in the other task. Intuitively, it is appealing to take a significant RT effect in the search task and a sensitivity not significantly above chance in the explicit recognition task as evidence for a difference. But this is a well-known misinterpretation of significance tests (Cantor , 1956; Appendix B in Franz & Gegenfurtner, 2008; Nieuwenhuis et al., 2011; Smith , 2020). Interpreting a non-significant result in the explicit recognition task is especially problematic because this task is often heavily underpowered due to fewer trials than the search task (Schlagbauer et al. , 2012; Vadillo et al. , 2016). Then, it should come as no surprise that—even with the same sensitivity underlying both tasks—the explicit recognition task fails to reach significance. The statistical power is just not high enough to reliably detect above-zero sensitivity. When sufficient statistical power is employed, studies do find non-negligible explicit recognition sensitivity (e.g., Colagiuri & Livesey, 2016; Zhao & Ren, 2020; Vadillo et al. , 2016).

However, the problem is deeper rooted than in a misinterpretation of null results in Frequentist statistics. Bayes factors (Morey & Rouder, 2011; Rouder et al. , 2009; Schönbrodt & Wagenmakers, 2018) suffer an analoguous problem (Palfi & Dienes, 2020): One Bayes factor supporting the alternative hypothesis in the search task and another Bayes factor supporting the null hypothesis in the explicit recognition task is not good enough evidence for an underlying difference between these two tasks.

This is especially true when taking into account that measures in the two tasks are on different scales—continuous RTs in the search task and binary predictions in the explicit recognition task. An ideal observer that had access to the continuous RTs (analogous to the baby weights in our example), would have to dichotomize these responses when employing this information to give a response in the explicit recognition task (Berry et al. , 2006). Thus, even if participants had some underlying continuous sense of how sure they are in recognizing repeated vs. new configurations, the binary response format forces them to discard this information. This entails a loss of test power in Frequentist statistics (Cohen , 1983) but also a systematic decrease of Bayes factors.

Additionally, there is another known problem of Bayes factors when it comes to small effect sizes. The issue is known as the catch-up phenomenon (Erven et al., 2012; Tendeiro & Kiers, 2019). For fixed, small effects (weak sensitivities), Bayes factors support the null hypothesis in small samples. But for sufficiently large samples, the same small effect results in Bayes factors supporting the alternative hypothesis. This creates a serious problem when different number of trials are used: The same underlying information will be evaluated differently, as support for the null hypothesis in one task with few observations and as support for the alternative hypothesis in another task with many observations (Fig. 8 in Tendeiro & Kiers, 2019).

A short simulation reveals these problems: We simulate $N=15$ participants each with a true sensitivity of $d'_i$ drawn from a normal distribution with mean 0.25 and standard deviation 0.3 (this corresponds to our assumption on $q^2 = 0.3^2 = 0.09$, see above). Each participant performs $K=96$ trials split evenly into the two conditions, repeated vs. new. Responses in these trials are sampled from normal distributions with expected value 0 for repeated and $d'_i$ for new conditions. Standard deviations of these two normal distributions are set to 1. For the search task, we compute the mean difference in responses from each participant based on all $K = K_\text {search} = 96$ trials. For the explicit recognition task, we only take the first 12 trials per condition for a total of $K_\text {explicit} = 24$ trials, which is a common number of trials for the explicit recognition task. We measure the sensitivity by computing hit and false alarm rates using the optimal decision criterion (median split) at $d'_i/2$.

Based on the individual differences and sensitivities, we used default Bayes factor analysis to evaluate the results (the ttestBF() function with default parameters from the R package BayesFactor, Morey et al., 2015). Across 1000 simulations, the Bayes factors of the search task were larger than in the explicit recognition task: The median Bayes factors were in the search task $BF_{10} = 3.9$ vs. in the direct task $BF_{01} = 0.9$. Thus, even an ideal observer with the exact same underlying information in the two tasks would be evaluated differently by Bayesian statistics due to the different scale and number of trials. These effects are exacerbated for smaller sensitivities.

A separate simulation in which the number of trials was held constant, $K_\text {search} = K_\text {explicit} = 96$, revealed that the dichotomization necessary for the binary response format still reduces BFs. Median Bayes factors in this case are $BF_{10} = 3.9$ in the search task (same as before) vs. $BF_{10} = 2.6$ in the explicit recognition task: The binary response format in the explicit recognition task discards information much like a dichotomization does when researchers apply it to their data (Cohen , 1983; MacCallum et al., 2002). Only here, the dichotomization is hidden in the response format and done by the participant internally. Nevertheless, this creates a difference in evaluating the two tasks no matter which statistical framework is used.

Thus, Bayes factors also evaluate the two tasks differently even though, by design of our simulation, we used the exact same information underlying responses in both tasks. The solution to this problem is straightforward: A comparative question, such as the question whether an ITA exists, must be answered *not* by statistics computed for the two tasks separately but by testing for a difference—by conducting a sensitivity comparison.

## Code Availability

The analysis scripts used in the current study are available in the Open Science Framework (https://osf.io/9rhs2). This does not include the original data sets shared by the authors of the five studies we reanalyzed.
