# Supplementary material for: No evidence for contextual cueing beyond explicit recognition
Source: Psychon Bull Rev. 2023 Oct 16;31(3):907–30. doi: 10.3758/s13423-023-02358-3 (PMC11192686; doi:10.3758/s13423-023-02358-3)
Supplement: Supplementary file 1 — (pdf 332 KB) [file 13423_2023_2358_MOESM1_ESM.pdf]

Supplementary Materials

No Evidence for Contextual Cueing Beyond Explicit Recognition

by S. Meyen, M. A. Vadillo, U. v. Luxburg, & V. H. Franz

Supplement A. Study Details 2

Supplement B. Learned Display Analysis 15

Supplement A Study Details

We here describe the reanalyzed studies individually. We show numerical results from our reanalysis in a tabular form.

- We indicate by the labels ***full data*** or ***reported statistics*** studies that we reanalyzed based on the full trial-by-trial data and studies that we reanalyzed based only on reported summary statistics, respectively.
- We indicate by the label ***inferred implicit*** whether a study infers implicit recognition and we provide quotes for these interpretations.
- We explain which experiments and conditions of the studies we used in our reanalysis as well as which we omitted and why.
- We describe which blocks or epochs we used for the reanalysis of the search task.

Chun and Jiang (1998, CJ-1998; reported statistics, inferred implicit). This seminal study presents six experiments. Only in Experiment 2 and 5, an explicit recognition task was administered. From these experiments, it is inferred that cueing was implicit ("The results of the recognition test and questioning support the hypothesis that contextual cueing is driven by incidental learning of implicit memory representations for configurations. Mean accuracy in the explicit recognition task was 52%.", p. 44). In Experiment 2, we reanalyzed the last (sixth) epoch. In Experiment 5, we reanalyzed the fourth epoch because this is the last epoch of the speeded task. After this task, the experiment switched to the accuracy task and RT effects became much smaller (76 ms effect in Epoch 4 vs. 11 ms and 15 ms in Epochs 5 and 6, respectively).

	Search Task			Explicit Recognition Task			Sensitivity Comparison		
	N	K	$d' \pm SE$	N	K	$d' \pm SE$	$d'_{\text{diff}} \pm SE$	CI 95%	BF_{10}
Exp. 2	14	96	0.24 ± 0.11	14	24	0.11 ± 0.16	0.13 ± 0.20	[-0.29, 0.55]	0.47
Exp. 5	18	120	0.19 ± 0.09	18	24	0.13 ± 0.14	0.06 ± 0.17	[-0.29, 0.41]	0.32

Chun and Phelps (1999, CP-1999; reported statistics, inferred implicit). The study presents results from patients with amnesia, a control group and secondary control group with increased task difficulty. We only reanalyzed results from the control group because patients with amnesia did not show a search task effect and the secondary control consisted only of five participants. We reanalyzed RT effects from Epochs 3 and 4 as this was defined as the contextual learning by the study and only this RT effect was reported (p. 845, bottom left). The study infers that cueing was implicit ("An important feature of our task is that memory traces for contextual information were not accessible to conscious awareness, and hence could be classified as implicit.", p. 844).

	Search Task			Explicit Recognition Task			Sensitivity Comparison		
	N	K	$d' \pm SE$	N	K	$d' \pm SE$	$d'_{\text{diff}} \pm SE$	CI 95%	BF_{10}
control	10	120	0.44 ± 0.17	10	24	0.14 ± 0.19	0.30 ± 0.25	[-0.26, 0.87]	0.95

Colagiuri et al. (2011, CLH-2011; reported statistics, inferred implicit). The study presents an experiment with multiple conditions but results were pooled because the explicit recognition task was also only reported across these conditions. Upon request (June 7, 2021), the full trial-by-trial data was not available (as of February 2022) and a suitable t test statistic for the search task was missing. Thus, we digitized their Figure 3 where RT effects are averaged across blocks and we then pooled these conditions as well. The study infers that cueing was implicit ("The most interesting result of this study was that information provided to participants [...] influenced implicit learning.", p. 404) mainly due to the close to chance sensitivity in the explicit recognition task, $d'_{\text{direct}} = 0.01$. However, strikingly, our reanalysis shows that the search task sensitivity was equally low ($d'_{\text{indirect}} = 0.04$).

Search Task			Explicit Recognition Task			Sensitivity Comparison		
N	K	$d' \pm SE$	N	K	$d' \pm SE$	$d'_{\text{diff}} \pm SE$	CI 95%	BF_{10}
464	320	0.04 ± 0.01	464	16	0.01 ± 0.03	0.03 ± 0.04	[-0.04, 0.10]	0.11

Colagiuri and Livesey (2016, CL-2016; full data, inferred implicit). The study presents three experiments with a remarkable sample size. We reanalyzed these experiments based on the full trial-by-trial data which the authors shared (February 23, 2022) upon request (June 7, 2021). We reanalyzed the last (twelfth) epoch in the search tasks. The study infers cueing to be implicit mainly from a correlation-based argument ("The critical theoretical and empirical analysis pertains to the relationship between cuing and recognition", p. 1997).

	Search Task			Explicit Recognition Task			Sensitivity Comparison		
	N	K	$d' \pm SE$	N	K	$d' \pm SE$	$d'_{\text{diff}} \pm SE$	CI 95%	BF_{10}
Exp. 1	63	32	0.16 ± 0.05	63	32	0.25 ± 0.07	-0.08 ± 0.08	[-0.24, 0.07]	0.07
Exp. 2	84	32	0.27 ± 0.05	84	64	0.15 ± 0.04	0.11 ± 0.06	[-0.01, 0.24]	1
Exp. 3	766	32	0.21 ± 0.02	766	32	0.17 ± 0.02	0.05 ± 0.03	[-0.00, 0.10]	0.45

Conci and von Mühlenen (2011, CM-2011; full data, inferred implicit). The study presents seven experiments which the authors shared (February 2, 2022, and October 28, 2022) upon request (January 26, 2022, and October 21, 2022). In all experiments, there were two conditions, one standard display and one variant (e.g., with varied colors or sizes in the displays). Only in Experiment 3, the two conditions needed to be reanalyzed jointly due to the design. The study infers cueing to be implicit based on the standard reasoning ("this nonsignificant difference between hits and false alarm rates indicates that no explicit awareness of the display repetitions was evident", p. 225).

	Search Task			Explicit Recog. Task			Sensitivity Comparison		
	N	K	$d' \pm SE$	N	K	$d' \pm SE$	$d'_{\text{diff}} \pm SE$	CI 95%	BF_{10}
Exp. 1 (color)	14	55	0.25 ± 0.08	14	12	0.20 ± 0.19	0.05 ± 0.21	[-0.41, 0.51]	0.33
Exp. 1 (standard)	14	52	0.76 ± 0.12	14	12	0.08 ± 0.22	0.68 ± 0.25	[0.14, 1.22]	7.1
Exp. 2 (size)	14	57	-0.00 ± 0.09	14	12	0.40 ± 0.19	-0.40 ± 0.20	[-0.83, 0.02]	0.11
Exp. 2 (standard)	14	56	0.64 ± 0.08	14	12	0.28 ± 0.17	0.36 ± 0.21	[-0.09, 0.82]	1.6
Exp. 3 (combined)	14	111	0.29 ± 0.07	14	24	0.26 ± 0.15	0.03 ± 0.15	[-0.29, 0.34]	0.31
Exp. 4 (uniform)	14	55	0.40 ± 0.17	14	12	-0.05 ± 0.16	0.45 ± 0.25	[-0.08, 0.99]	1.9
Exp. 4 (variable)	14	56	0.39 ± 0.13	14	12	0.21 ± 0.33	0.19 ± 0.33	[-0.52, 0.90]	0.44
Exp. 5 (nonsalient)	14	52	0.90 ± 0.10	14	24	0.28 ± 0.12	0.62 ± 0.13	[0.34, 0.90]	170
Exp. 5 (salient)	14	48	0.21 ± 0.08	14	24	0.28 ± 0.12	-0.07 ± 0.16	[-0.41, 0.27]	0.2
Exp. 6 (size)	14	54	0.01 ± 0.09	14	12	0.37 ± 0.27	-0.35 ± 0.27	[-0.93, 0.22]	0.13
Exp. 6 (standard)	14	54	0.91 ± 0.10	14	12	-0.06 ± 0.16	0.97 ± 0.20	[0.55, 1.39]	250
Exp. 7 (size)	14	54	0.14 ± 0.14	14	12	0.13 ± 0.21	0.01 ± 0.25	[-0.54, 0.56]	0.28
Exp. 7 (standard)	14	54	0.96 ± 0.12	14	12	-0.08 ± 0.23	1.03 ± 0.28	[0.44, 1.63]	37

Dixon et al. (2010, DZR-2010; reported statistics, inferred implicit). The study reported two experiments (Experiment 1 and 1A) with children as participants. We only reanalyzed Experiment 1A because Experiment 1 did not apply an explicit recognition task. Repeated vs. new configurations were not mixed in the last block but Block 6 had repeated and Block 7 had new stimulus configurations. We therefore reanalyzed the difference between these two blocks. Note that the study reports an F value for the paired test between these blocks, for which the corresponding t value is $t = +\sqrt{F}$. (This transfer from F to t is not possible in most other studies because other ANOVA designs typically include other factors.) The study concluded that cueing was implicit from a correlation-based argument ("Importantly, individual differences in children's level of awareness of the task structure were not related to the magnitude of the contextual cueing effect", p. 166).

	Search Task			Explicit Recognition Task			Sensitivity Comparison		
	N	K	$d' \pm SE$	N	K	$d' \pm SE$	$d'_{\text{diff}} \pm SE$	CI 95%	BF_{10}
Exp. 1A	12	16	0.41 ± 0.20	12	8	0.47 ± 0.27	-0.06 ± 0.33	[-0.79, 0.67]	0.25

Geringswald et al. (2012, GBP-2012; reported statistics). The study presents an experiment in impaired and unimpaired viewing conditions: Impaired viewing conditions were measured in healthy participants where the visual field was distorted to simulate a central scotoma based on online eye-tracking of the participants. We reanalyzed the last (fourth) epoch of the search task. The study did not report a t statistic for this but we obtained a measure for the standard error based on the reported mean effects in their Table 1, a digitization from their Figure 2, and their reported F statistics. The study did not infer cueing to be implicit.

	Search Task			Explicit Recognition Task			Sensitivity Comparison		
	N	K	$d' \pm SE$	N	K	$d' \pm SE$	$d'_{\text{diff}} \pm SE$	CI 95%	BF_{10}
unimpaired	25	120	0.26 ± 0.08	25	24	0.36 ± 0.12	-0.10 ± 0.14	[-0.40, 0.19]	0.13
impaired	25	120	0.03 ± 0.07	25	24	0.17 ± 0.12	-0.14 ± 0.14	[-0.43, 0.14]	0.11

Geringswald et al. (2013, GHHP-2013; reported statistics). The study presents one experiment with different viewing conditions (monocular, binocular and a control condition). We reanalyzed the last (fourth) epoch of the search task. The study did not infer cueing to be implicit.

	Search Task			Explicit Recognition Task			Sensitivity Comparison		
	N	K	$d' \pm SE$	N	K	$d' \pm SE$	$d'_{\text{diff}} \pm SE$	CI 95%	BF_{10}
monocular	16	120	0.35 ± 0.11	16	24	0.29 ± 0.15	0.06 ± 0.19	[-0.34, 0.45]	0.33
control	16	120	0.23 ± 0.10	16	24	0.34 ± 0.15	-0.11 ± 0.18	[-0.49, 0.27]	0.17
binocular	16	120	0.25 ± 0.10	16	24	-0.03 ± 0.15	0.28 ± 0.18	[-0.11, 0.66]	1.2

Kawahara (2003, K-2003; reported statistics, inferred implicit). The study reported four experiments (1A, 1B, 2A and 2B) of which we could only reanalyze Experiment 2A because the others did not report the necessary statistics: For Experiment 2A, an additional analysis was reported on p. 850 from which we used the t value. We thereby reanalyzed the last (sixth) epoch from the search task. The study infers cueing to be implicit based on the standard reasoning (“The results of Experiment 2A and 2B [low sensitivity in the recognition task] showed that the observers were able to acquire the context of layouts including 3D structure implicity”, p. 849).

	Search Task			Explicit Recognition Task			Sensitivity Comparison		
	N	K	$d' \pm SE$	N	K	$d' \pm SE$	$d'_{\text{diff}} \pm SE$	CI 95%	BF_{10}
Exp. 2A	14	120	0.28 ± 0.10	17	24	0.09 ± 0.14	0.19 ± 0.18	[-0.18, 0.57]	0.71

Manns and Squire (2001, MS-2001; reported statistics, inferred implicit). The study presents data from patients with lesions to hippocampal regions (H+), patients with lesions to medial temporal regions (MTL+) as well as healthy controls. We reanalyzed search task effects averaging blocks 3–8 because this is what the study reported as cueing effects. The study infers cueing to be implicit based on the standard reasoning (“Both groups (H+ and CON-1) performed at chance overall on the yes-no recognition test given after block 4 as well as on the test given at the end of the session (mean percent correct ± SEM = 51.7 ± 3% and 51.7 ± 2% for CON-1; and 50.8 ± 2% and 51.7 ± 2% for the amnesic patients; one-sample t-tests, all $P > 0.1$)”, p. 780).

	Search Task			Explicit Recognition Task			Sensitivity Comparison		
	N	K	$d' \pm SE$	N	K	$d' \pm SE$	$d'_{\text{diff}} \pm SE$	CI 95%	BF_{10}
H+	5	720	0.38 ± 0.25	5	24	0.09 ± 0.26	0.29 ± 0.37	[-0.73, 1.31]	0.76
CON1	15	720	0.23 ± 0.09	15	24	0.09 ± 0.15	0.14 ± 0.18	[-0.25, 0.52]	0.51
CON2	8	720	0.34 ± 0.16	8	24	0.22 ± 0.21	0.12 ± 0.26	[-0.50, 0.74]	0.48

Rausei et al. (2007, RMJ-2007; reported statistics, inferred implicit). The study presents two experiments of which we could only reanalyze Experiment 2 because no paired t tests were reported in Experiment 1 but only in Experiment 2. In this experiment, target-distractor similarity was varied (label both does not refer to averaging over the other two conditions we reanalyzed but to the "both-repeated" condition in the study). We reanalyzed results from the transfer phase which followed the training phase in the experiment. Cueing was inferred to be implicit ("Finally, there was no evidence for explicit awareness of the repetition. Hit rate for correctly identifying a repeated display (55%) was not significantly different from the false alarm rate for misidentifying an unrepeated display (52%), t(39) < 1", p. 1326).

	Search Task			Explicit Recognition Task			Sensitivity Comparison		
	N	K	$d' \pm SE$	N	K	$d' \pm SE$	$d'_{\text{diff}} \pm SE$	CI 95%	BF_{10}
Exp. 2 (both)	40	64	0.28 ± 0.07	40	32	0.08 ± 0.08	0.20 ± 0.11	[-0.03, 0.42]	1.4
Exp. 2 (similar)	40	32	0.13 ± 0.08	40	32	0.02 ± 0.08	0.10 ± 0.11	[-0.13, 0.33]	0.4
Exp. 2 (dissimilar)	40	32	0.19 ± 0.08	40	32	0.00 ± 0.08	0.19 ± 0.11	[-0.04, 0.43]	1.2

Schankin and Schubö (2009, SS-2009; reported statistics, inferred implicit). The study presents one experiment and we reanalyzed the last three epochs of the search task because the study reports RT effects from these arguing that cueing effects are comparable. The study infers cueing to be implicit based on the standard reasoning ("Similarly to previous studies, the knowledge about repeated configurations was implicit. In the recognition test, the hit rate (49%) did not differ significantly from the false alarms rate (43%)", p. 672).

Search Task			Explicit Recognition Task			Sensitivity Comparison		
N	K	$d' \pm SE$	N	K	$d' \pm SE$	$d'_{\text{diff}} \pm SE$	CI 95%	BF_{10}
14	576	0.14 ± 0.09	14	32	0.15 ± 0.14	-0.01 ± 0.17	[-0.38, 0.35]	0.26

Schankin et al. (2008, SSS-2008; reported statistics, inferred implicit). The study reports one experiment which we reanalyzed based on reported statistics from the last three epochs as defined to be the contextual cueing effect by the study. The study infers cueing to be implicit ("Similarly to previous studies, the knowledge about repeated configurations was implicit. In the recognition test, the hit rate (42%) did not differ significantly from the false alarms rate (36%)", p. 101).

Search Task			Explicit Recognition Task			Sensitivity Comparison		
N	K	$d' \pm SE$	N	K	$d' \pm SE$	$d'_{\text{diff}} \pm SE$	CI 95%	BF_{10}
16	576	0.39 ± 0.11	16	32	0.16 ± 0.13	0.23 ± 0.17	[-0.14, 0.60]	0.93

Vadillo et al. (2022, VMLDS-2022; full data). The study presents four experiments mainly discussing correlation-based arguments. The authors shared their data early on in the project (June 22, 2021) and it is openly accessible at `https://osf.io/fuzvn/`. We reanalyzed Experiments 1–3 because Experiment 4 used a non-binary explicit recognition task. We used the last (twelfth) epoch for our reanalyses. The study did not infer cueing to be implicit.

	Search Task			Explicit Recognition Task			Sensitivity Comparison		
	N	K	$d' \pm SE$	N	K	$d' \pm SE$	$d'_{\text{diff}} \pm SE$	CI 95%	BF_{10}
Exp. 1	104	32	0.24 ± 0.05	104	32	0.29 ± 0.06	-0.05 ± 0.07	[-0.19, 0.09]	0.07
Exp. 2	110	32	0.21 ± 0.04	110	64	0.31 ± 0.04	-0.10 ± 0.06	[-0.22, 0.02]	0.04
Exp. 3	122	32	0.26 ± 0.04	122	64	0.18 ± 0.03	0.08 ± 0.05	[-0.02, 0.19]	0.6

Xie et al. (2020, XCZ-2020; full data, inferred implicit). The study presents four experiments. The authors laudably made their data openly accessible and we acquired it without contacting the authors (November 22, 2021). Only the last experiment had an explicit recognition task to be reanalyzed. Nevertheless, we analyzed search tasks from Experiments 1–3 for estimating the RT variance ratios (Figure 3). We reanalyzed the last (tenth) epoch of the search tasks. The study inferred cueing to be implicit ("post-experimental recognition tests revealed participants' ability to distinguish repeated from novel conditions only to be at chance level, indicating that contextual cueing is mediated by implicit memory representations.", p. 11).

	Search Task			Explicit Recognition Task			Sensitivity Comparison		
	N	K	$d' \pm SE$	N	K	$d' \pm SE$	$d'_{\text{diff}} \pm SE$	CI 95%	BF_{10}
Exp. 4	15	84	0.10 ± 0.08	15	24	0.15 ± 0.19	-0.04 ± 0.22	[-0.51, 0.42]	0.23

Zang et al. (2016, ZGAMS-2016; reported statistics, inferred implicit). The study reports three experiments. During training, an upwards pointing cuboid was presented in the background. In Epoch 5, the cuboid was pointing downward and in Epoch 6 no cuboid was presented, which are the two epochs we reanalyzed. In Experiment 2, a high number of participants (6 out of 16, 38%) was excluded due to high explicit recognition performance. Therefore, this experiment suffered from severe regression to the mean issues and we did not reanalyze it. The other two experiments only excluded one participant each so that regression to the mean was not as severe. The study inferred cueing to be implicit based on the standard reasoning and a correlation-based argument ("[...] mean hit rates [...] were numerically higher than the false alarm rates [...]. However, these differences were not significant [...] Thus, taken together, there was no evidence that contextual cueing in the current experiment was based on explicit memory of old displays.", p. 8).

	Search Task			Explicit Recognition Task			Sensitivity Comparison		
	N	K	$d' \pm SE$	N	K	$d' \pm SE$	$d'_{\text{diff}} \pm SE$	CI 95%	BF_{10}
Exp. 1 (down)	10	32	0.01 ± 0.15	10	16	0.02 ± 0.22	-0.01 ± 0.27	[-0.61, 0.60]	0.3
Exp. 1 (without)	10	32	0.19 ± 0.16	10	16	0.30 ± 0.22	-0.11 ± 0.27	[-0.72, 0.50]	0.24
Exp. 3 (down)	15	32	0.26 ± 0.13	15	16	0.25 ± 0.18	0.01 ± 0.22	[-0.47, 0.49]	0.27
Exp. 3 (without)	15	32	0.39 ± 0.15	15	16	-0.05 ± 0.18	0.44 ± 0.23	[-0.05, 0.94]	2.1

Zang et al. (2015, ZJMS-2015; reported statistics). The study presents three experiments in which participants started the search task with tunnel vision (they could not see the whole stimulus configuration at once) and only later they were tested in a free viewing condition. We reanalyzed results only from this last (sixth) epoch with free viewing consistent with the study inferring that contextual cueing only shows at that stage.

	Search Task			Explicit Recognition Task			Sensitivity Comparison		
	N	K	$d' \pm SE$	N	K	$d' \pm SE$	$d'_{\text{diff}} \pm SE$	CI 95%	BF_{10}
Exp. 1	13	80	0.48 ± 0.15	13	24	0.39 ± 0.16	0.09 ± 0.22	[-0.40, 0.57]	0.38
Exp. 2	13	80	0.33 ± 0.13	13	24	0.20 ± 0.16	0.13 ± 0.21	[-0.32, 0.59]	0.48
Exp. 3	13	80	0.24 ± 0.12	13	24	0.22 ± 0.16	0.02 ± 0.20	[-0.42, 0.47]	0.31

Zhao and Ren (2020, ZR-2020; full data). The study presents three experiments and we were able to reanalyze all of them based on the full trial-by-trial data which the authors shared swiftly (December 22 2020) upon request (December 19 2020). Experiments 1 and 2 varied contrast of the presented stimuli and we reanalyzed these conditions separately. In these experiments, we reanalyzed the last (seventh) epoch of the search task. The study abstains from making strong claims about the implicit nature of cueing.

	Search Task			Explicit Recognition Task			Sensitivity Comparison		
	N	K	$d' \pm SE$	N	K	$d' \pm SE$	$d'_{\text{diff}} \pm SE$	CI 95%	BF_{10}
Exp. 1 (high)	37	32	-0.19 ± 0.07	37	8	0.20 ± 0.14	-0.39 ± 0.15	[-0.70, -0.08]	0.05
Exp. 1 (low)	37	32	0.49 ± 0.08	37	8	0.22 ± 0.13	0.27 ± 0.15	[-0.04, 0.58]	1.4
Exp. 2 (high)	31	32	-0.02 ± 0.07	31	8	0.24 ± 0.14	-0.26 ± 0.16	[-0.60, 0.07]	0.08
Exp. 2 (low)	31	32	0.35 ± 0.06	31	8	0.25 ± 0.14	0.10 ± 0.15	[-0.21, 0.40]	0.34
Exp. 3	33	32	0.16 ± 0.07	33	8	0.57 ± 0.12	-0.40 ± 0.11	[-0.63, -0.18]	0.05

Zellin et al. (2011, ZCVM-2011; reported statistics, inferred implicit). The study presents three experiments of which the reported statistics only allowed to reanalyze Experiment 2. There were two conditions in which either one or two target stimuli were presented in each stimulus display. We were only able to reanalyze the two-target stimulus displays because only for these the necessary statistics were reported (see p. 2069, right column). We reanalyzed the last epoch of this search task. The study infers cueing to be implicit based on the standard reasoning ("the numbers of hits (57.9%) and false alarms (49.6%) were statistically comparable for two-target displays, $t(20) = 1.84$, $p = .08$, suggesting that participants were mostly unable to explicitly discern between old and new contexts.", pp. 2069–2070).

	Search Task			Explicit Recognition Task			Sensitivity Comparison		
	N	K	$d' \pm SE$	N	K	$d' \pm SE$	$d'_{\text{diff}} \pm SE$	CI 95%	BF_{10}
Exp. 2 (two)	21	120	0.14 ± 0.08	21	24	0.17 ± 0.13	-0.03 ± 0.15	[-0.35, 0.29]	0.2

Zellin et al. (2013, ZCMM-2013; reported statistics, inferred implicit). The study reports Experiments 1A, 1B, 2A, 2B, 3 and 4 of which we reanalyzed all expect Experiments 1B and 2B because in these experiments, the statistics from a paired t test that we needed for our reanalysis were not reported. For the other experiments, we reanalyzed the last epoch of the search task in each experiment. Note that this study excluded participants that showed a negative cueing effect potentially incurring a regression to the mean problem. The study inferred cueing to be implicit from a correlation-based argument.

	Search Task			Explicit Recognition Task			Sensitivity Comparison		
	N	K	$d' \pm SE$	N	K	$d' \pm SE$	$d'_{\text{diff}} \pm SE$	CI 95%	BF_{10}
Exp. 1A	12	120	0.42 ± 0.14	12	24	0.49 ± 0.17	-0.07 ± 0.22	[-0.56, 0.42]	0.23
Exp. 2A	12	120	0.43 ± 0.15	12	24	-0.02 ± 0.17	0.45 ± 0.23	[-0.04, 0.95]	2.5
Exp. 3	14	120	0.27 ± 0.11	14	24	0.27 ± 0.16	0.00 ± 0.19	[-0.41, 0.42]	0.27
Exp. 4	14	120	0.32 ± 0.12	14	24	0.04 ± 0.16	0.28 ± 0.20	[-0.14, 0.71]	1.1

Supplement B Learned Display Analysis

In the contextual cueing paradigm, participants typically learn only a subset of the repeated stimulus displays (Geyer et al., 2010, 2020; Smyth & Shanks, 2008). Inspired by a suggestion of a reviewer, we tested for ITAs in this subset of displays.

For this, we reanalyzed the data from Experiment 3 in Colagiuri and Livesey (2016) by first subselecting the learned displays and then conducting the same sensitivity comparison as before. The subselection was performed in two different ways (see B1 and B2 below). Both were proposed by Smyth and Shanks (2008) who previously only used them for counting the number of learned displays. But because we conduct an analysis after the selection, a problem with regression to the mean arises from this procedure (Shanks, 2017). We will discuss the problems below.

B1 Selecting Learned Displays by Large RT Effects

The first criterion considers a repeated stimulus display as learned if the mean RT of this display falls below the 99% confidence interval around the mean RT of non-repeated displays (Smyth & Shanks, 2008). We selected the displays in the Epochs 9–11 and evaluated, as before, the ITA results based on the last epoch, Epoch 12. This analysis yielded a clear ITA: Indirect sensitivities ($M = 0.58$, $SE = 0.03$) were clearly larger than than direct sensitivities ($M = 0.03$, $SE = 0.03$) with an average difference of $M = 0.55$ ($SE = 0.04$), $t(603) = 12.7$, $p < .001$, 95% CI $[0.46, 0.63]$, $BF_{10} > 1000$. The lower number of degrees of freedom comes from the fact that some participants did not learn any displays according to this criterion.

However, this result suffers from a regression to the mean artifact. The selected displays already produced fast RTs when participants encountered them for the very first time: Repeated displays later classified as learned produced faster RTs already when shown for the first time ($M = 1485$ ms, $SD = 580$) compared to new displays ($M = 1615$ ms, $SD = 406$) by $M = 130$ ms ($SD = 574$), $t(603) = 5.56$, $p < .001$. This indicates that the sensitivity difference does not stem from learning but simply from the selected displays being easier and a priori faster to respond to. By selecting displays this way, the RT difference is confounded by features of the displays and does not

necessarily reflect recognition.

B2 Selecting Learned Displays by High Explicit Accuracy

The second criterion considers a repeated stimulus display as learned if it was correctly classified as repeated in more than half of the trials during the explicit recognition task (Smyth & Shanks, 2008). Since there was only one explicit recognition block, we selected the displays based on this block and then evaluated the ITA based on it as well. This analysis yielded clear evidence for the *opposite* of an ITA. Indirect sensitivities ($M = 0.19$, $SE = 0.02$) were clearly lower than direct sensitivities ($M = 1.63$, $SE = 0.02$) with a difference of $M = -1.44$ ($SE = 0.03$), $t(741) = -49.3$, $p < .001$, 95% CI $[-1.5, -1.4]$, $BF_{10} < 1/1000$. Clearly, this approach suffers from double dipping the data: We first select displays with a good explicit recognition capability and then find them to have a relatively high sensitivities compared to the search task sensitivities, which is to be expected by the selection procedure.

Conclusion

Both approaches come with the methodological problem of regression to the mean due to post hoc selection. Thus, further methodological work — for example better control conditions or experimental manipulation of learning — is required to apply the sensitivity comparison to selected displays.
